# Aberrant enterocyte progenitor clustering as an early life biomarker of *Drosophila* aging

**DOI:** 10.1016/j.isci.2025.111967

**Published:** 2025-02-06

**Authors:** Constantina Neophytou, Savvas Teloni, Maria Koumouri, Marine Stefanutti, Panagiota Gianni, Vural Yilmaz, Katerina Strati, Yiorgos Apidianakis

**Affiliations:** 1 Department of Biological Sciences, University of Cyprus, 2109 Nicosia, Cyprus; 2 Genetics and Developmental Biology Department, Institut Curie, PSL Research University, Sorbonne University, CNRS UMR 3215, INSERM U934, 75248 Paris, France

**Keywords:** Cell biology, Stem cells research

## Abstract

Stem cell accumulation and mutation-derived tumors are two hallmarks of *Drosophila* midgut aging. They imply a decline in stem cell signaling homeostasis late in life and a robust homeostasis in young adults. Contrary to this, we find spontaneously developing stem-like cells that vary in size and ploidy, have a stem-enteroblast mixed identity, achieve higher mitotic rate per cell, exhibit DNA replication stress, and are inherently prone to clustering. Reduction of mitosis or DNA replication stress lessens the production of these cells but does not explain the loss of their proper differentiation. However, young enterocyte progenitors also display epigenetic plasticity in Notch signaling network genes and *Notch* locus instability. Strikingly, reinforcing Notch signaling in enteroblasts, alleviates dysplasia and extends overall survival and survival to infection. Thus, Notch signaling between prospective stem cells and enteroblasts is never sufficiently on, producing stem-enteroblast mixed identity cells that cluster and compromise homeostasis and overall aging.

## Highlights

• Notch signaling between progenitors is already flawed in young adults
• Young midguts contain aggregates of overproliferating mixed identity cells
• ISC-like cell aggregates compromise resilience to infection and overall aging
• ISC-like cell aggregates offer a treatable early life biomarker of old-age pathology

## Graphical abstract

**Figure:**
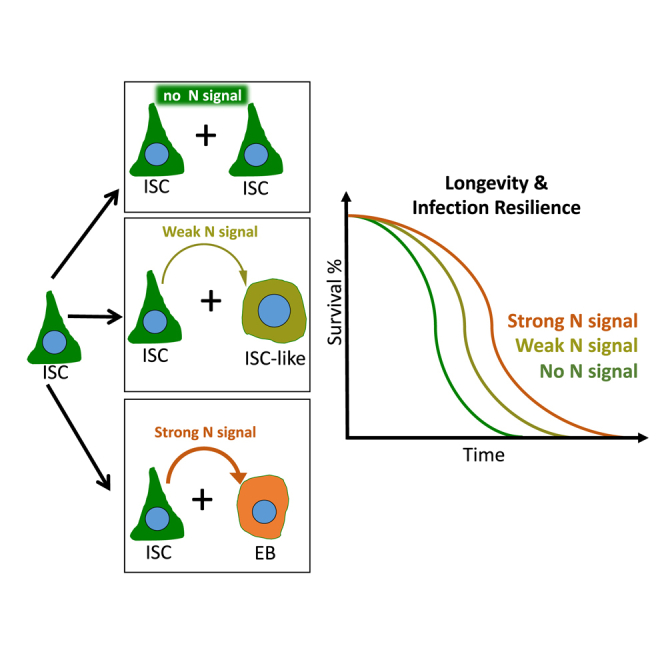

## Introduction

Regenerative capacity is essential for tissue integrity during growth, aging, infection, and stress.^1^ The adult *Drosophila* midgut similarly to its mammalian counterpart is rapidly self-renewing to keep up with the microbes, food, and metabolites it harbors.^2^ *Drosophila* midgut cell renewal depends on the alternating asymmetric to symmetric division mode of multipotent intestinal stem cells (ISCs).^3^ Asymmetric ISC divisions and daughter cell differentiation enable tissue renewal and tissue size maintenance by constantly replenishing dying cells, while symmetric ISC divisions enable the expansion of the ISC population and tissue growth upon tissue development, injury or physiologic adaptation.^3^^,^^4^

*Drosophila* midgut ISCs express Escargot (Esg) and Delta (Dl), the ligand of receptor Notch. ISC daughter cells either retain the ISC status or differentiate transiently into enteroblasts (EBs), which are marked with the Notch activity reporter, Su(H)GBE (Suppressor of Hairless and Grainyhead Binding Elements), or turn into pre-enteroendocrine cells (pre-EEs) that are positive for Prospero (Pros) and Dl.^5^^,^^6^^,^^7^^,^^8^ EBs terminally differentiate into polyploid absorptive enterocytes (ECs) that express Myosin 31DF (Myo1A) and nubbin (Pdm1), while pre-EEs differentiate into hormone-producing EEs that express Pros.^7^^,^^9^^,^^10^ During midgut homeostasis, ∼80% of ISCs divisions are asymmetric producing one ISC and one EB (∼70%) or one ISC and one pre-EE (∼10%). Pre-EEs may divide once producing EE doublets.^11^ The remaining ∼20% of ISC divisions are symmetric producing two ISCs (∼10%) or two EBs (∼10%).

During ISC to EB lineage specification, an interplay between Notch and Dpp/BMP signaling, the Par complex, integrins, Numb and Sara endosomes dictate ISC daughter cell asymmetric fates.^12^^,^^13^^,^^14^^,^^15^ As a result, Dl is retained by one of the two daughter cells, the prospective ISC, and the other daughter cell usually loses Dl expression and receives the Notch signal to adopt the polyploid EC fate.^16^ During ISC to EE lineage specification, the Par complex initially controls the asymmetric distribution of Pros in the pre-EE, which retains some Dl expression and sends a low-Notch signal to the prospective ISC securing its fate.^6^ During symmetric divisions, daughter cells segregate Dl symmetrically, but do not mount a high-Notch signal, due to high-Dpp/BMP signaling, giving rise to two ISCs. Alternatively, daughter cells exchange high-Notch signal, due to low-Dpp/BMP signaling, giving rise to two EBs.^17^

Midgut ISCs exhibit differences in their activity along the midgut.^18^^,^^19^^,^^20^^,^^21^ ISCs located in the posterior regions generally divide faster than those of the anterior and middle midgut.^18^ Regional ISC activity may be shaped by developmental,^22^ microbial,^23^^,^^24^ or nutritional cues and seemingly epigenetic regional gene expression.^21^ Interestingly, ISC daughter cells retain their regional expression pattern even when drifting into a neighboring compartment, indicating their epigenetic regulation.^18^ Moreover, female midgut ISCs are more mitotic than male ones due to a sex determination pathway during aging or upon injury,^25^ and are more prone to dysplasia.^26^^,^^27^^,^^28^

Aging is also associated with loss of lumen acidity, immunosenescence,^29^^,^^30^ dysbiosis, higher ROS production, and JNK signaling.^31^^,^^32^^,^^33^ Signaling through JNK promotes ISC mitotic spindle positioning parallel to the basement membrane, increased symmetric divisions, and midgut dysplasia.^34^ Despite this wealth of information, midgut aging has so far been studied by contrasting young and old flies making it unclear as to when or how the aging process starts. Moreover, aged stem cells exhibit loss of heterozygosity and DNA deletions and complex rearrangements leading to gene inactivation, most prominently of the X-linked gene *Notch*.^35^^,^^36^ This, in turn, leads to ISC tumor formation in old wild type males bearing only one copy of *Notch*. Tumors are not evident in young flies but develop over time in up to ∼10% of male midguts as large groups of ISCs and EEs of up to thousands of cells.^36^ What predisposes their formation is also unclear, although increased ISC proliferation and DNA damage response (DDR) may have an influence.^37^^,^^38^ DDR is controlled by the phosphorylation cascade in which histone H2avD, analogous to the mammalian H2aX.^39^ is phosphorylated (γH2av) by the ataxia telangiectasia mutated (ATM), the ATM- and Rad3-related (ATR) and the DNA-dependent protein kinase (DNA-PK) on a serine or threonine preceding a glutamine (pS/TQ).^40^ ATM and ATR are regulators of ISC maintenance and proliferation.^38^ Both of them are activated in response to double-strand breaks (DSBs), but ATR is also activated upon DNA replication stress (DRS).^38^ In response to ROS-associated DNA damage, aging ISCs develop γH2avD foci^37^ and induction of the ATM/ATR phosphorylation marker pS/TQ^38^^,^^41^ and 8-oxo-2′-deoxyguanosine.^37^ γH2av is phosphorylated by ATM/ATR by DSBs, replication fork collapse, metabolic stress and oncogene expression.^42^ In turn, γH2av induces DNA end processing enzymes working on DSB repair, including homologous recombination (HR) and non-homologous end-joining (NHEJ).^39^ However, accumulation of γH2av may also be triggered by DRS and ATR, independently of DSBs.^43^ DRS induces DNA repair genes, such as *spn-B*, slows DNA replication forks, and extends the S phase in response to nucleotide starvation.^44^^,^^45^ While DDR and presumably DRS is prominent upon aging and oxidative stress in *Drosophila* midgut ISCs,^37^ their impact on young ISCs remains elusive.

Epigenetic deregulation is another feature of aging.^46^^,^^47^ The *Drosophila* midgut ISCs exhibit moderate changes in chromatin organization over time, including a decline in H3K27 dimethylation (H3K27me2), adoption of the EE fate, and dysplasia.^48^ Trithorax (Trx) group factors, namely, Trx, Kismet, and Trx-related (Trr), inhibit ISC proliferation.^49^ Moreover, the chromatin state of ISC-enriched genes transitions more toward repression in EEs compared to ECs, indicating the relative plasticity of the latter.^50^ However, so far there is no evidence of a primary trigger or causal factor of *Drosophila* midgut aging. In this study, we provide clear evidence of mis-differentiated midgut stem-enteroblast cells very early in the adult life of wild type *Drosophila* contributing to the aging process. We performed ATAC-Seq revealing regional *Notch* locus epigenetic regulation and devised *NotchTSS-331tubGal80*, a tool that showed *Notch* locus epigenetic instability in ISCs and EBs. We also performed genetic and pharmacological experiments revealing a suboptimal Notch signaling in young midgut progenitors.

## Results

### ISCs start clustering primarily in the posterior midguts of young and seemingly healthy *Drosophila* adults

**Figure 1:**
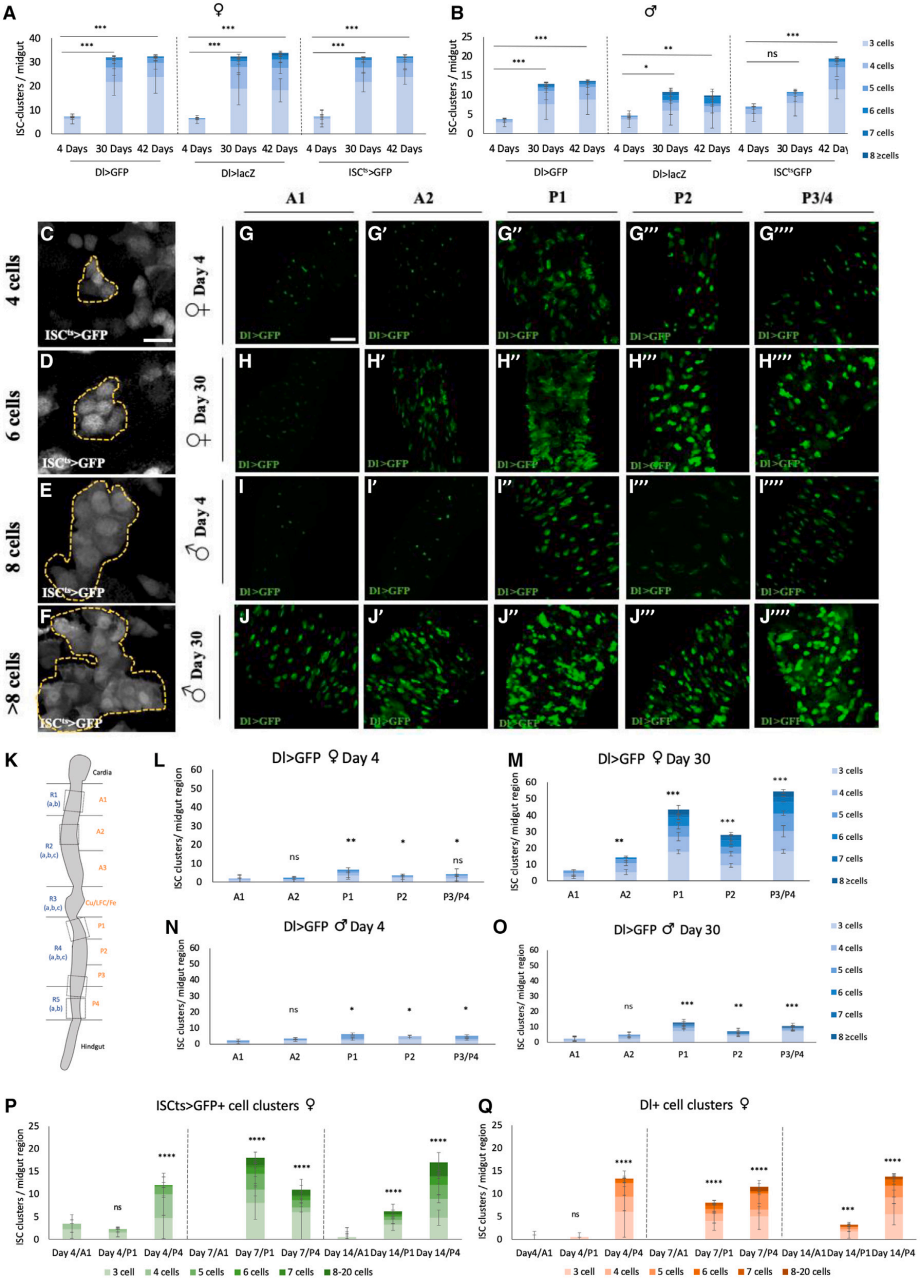
ISC-like clusters appear spontaneously in the female and male midgut of young adults and expand during aging (A-B) ISC-like cluster enumeration in the female (A) and male (B) midguts of *Dl-GFP, Dl-lacZ* and *ISC*^*ts*^*>GFP* fly strains outcrossed to Oregon-R and incubated for 4, 30 or 42 days at 25°C. (C–F) Representative *ISC*^*ts*^*>GFP*+ clusters of 4 (C), 6 (D), 8(E) and >8 cells(F) from day 7 females at 29°C. (G–J) Representative images of A1, A2 (‘), P1 (“), P2 (‘’’) and P3/P4 (“”) of *Dl-GFP* (green) flies. (G and H) Female midguts at day 4(G) and day30 (H) and (I-J) male midguts at day 4 (I) and day 30 (J) at 25°C. (K) Illustration of the midgut and its anteroposterior regions (in frames) assessed in this work. (L–O) Enumeration of ISC-like clusters in A1, A2, P1, P2 and P3/P4 of *Dl>GFP* female flies at day 4 (L) and 30 (M), at 25°C and male flies at day 4 (N) and 30 (O) at 25°C. (P-Q) Enumeration of ISC-like clusters in A1, P1, and P4 of female young (day 4 and 7 at 29°C) and moderately old (day 14 at 29°C) *ISC*^*ts*^*>GFP* (P) and Dl-stained (Q) fly midguts. Experiments were repeated at least twice. Scale bar: 50 μm for (C-F) and 75 μm for (G-J). Statistical significance was via chi-square test using *n* = 10 for (A-B) (L-O) and *n* = 4 for (P-Q). (A, B, L–O, P, Q) Chi-square test, expecting equal number of 3+ cell clusters between conditions, *n* = 10 midguts, 1° freedom, ∗*p* < 0.05, ∗∗*p* < 0.01, ∗∗∗*p* < 0.001, and ∗∗∗∗*p* < 0.0001.; ns, not statistically significant. See also Figure S1.

ISCs, usually identified by the expression of *esg-Gal4* and Dl, accumulate during aging and in tumor-prone flies ingesting pathogenic bacteria.^31^^,^^51^^,^^52^^,^^53^ Typical ISCs are negative for the Notch signaling reporter and EB marker, Su(H)GBE. However, the timing and trigger for their accumulation in old adults remains unclear. To assess ISC accumulation over time, we marked ISCs using three different fly lines: *ISC*^*ts*^*-Gal4 UAS-GFP* (*esg-Gal4 tub-Gal80*^*ts*^ *UAS-EGFP Su(H)GBE-tub-Gal80*),^8^ *Dl-lacZ*^54^ and *Dl-Gal4 UAS-GFP*^55^ outcrossed to wild-type Oregon-R flies at 25°C. In addition to the anticipated single and occasional double ISCs, we detected clusters of 3–16 ISC-like cells using all three ISC markers. Four-day-old females and males frequently exhibited clusters of 3–6 cells, while 30- and 42-day-old females and males exhibited approximately triple the number of clusters containing 3–16 cells (Figures 1A–1J). The three different ISC markers gave comparable results. Specifically, females exhibited up to 4 times, and males up to 3 times, the number of clusters in old versus young fly midguts (Figures 1A and 1B). In agreement with the sex bias in ISC activity,^25^^,^^28^ females exhibited triple the number of clusters compared to males (Figures 1A and 1B).

Using the *Dl-Gal4 UAS-GFP* line outcrossed to Oregon-R at 25°C we quantified clusters in the A1, A2, P1, P2, and around P3/P4 border (P3/P4) midgut regions of young and old flies of both sexes^18^^,^^19^ (Figures 1K–1O). Four-day-old adult females and males showed increased cluster formation in the posterior P1, P2 and P3/P4 regions compared to the anterior A1 region, while 30-day-old flies exhibited more clusters and more cells per cluster in the posterior compartment, predominantly in P1 and P3/P4 regions, compared to 4-day-old flies (Figures 1L–1O). We noticed similar regional and age differences when using the GFP as well as Dl antibody staining of *ISC*^*ts*^*-Gal4 UAS-GFP* line outcrossed to *w*^*1118*^ (Figures 1P and 1Q). Thus, the P1 and P3/P4 regions serve as “hotspots” for ISC-like cluster formation, whereas the A1 region as a “cold-spot”.

**Figure 2:**
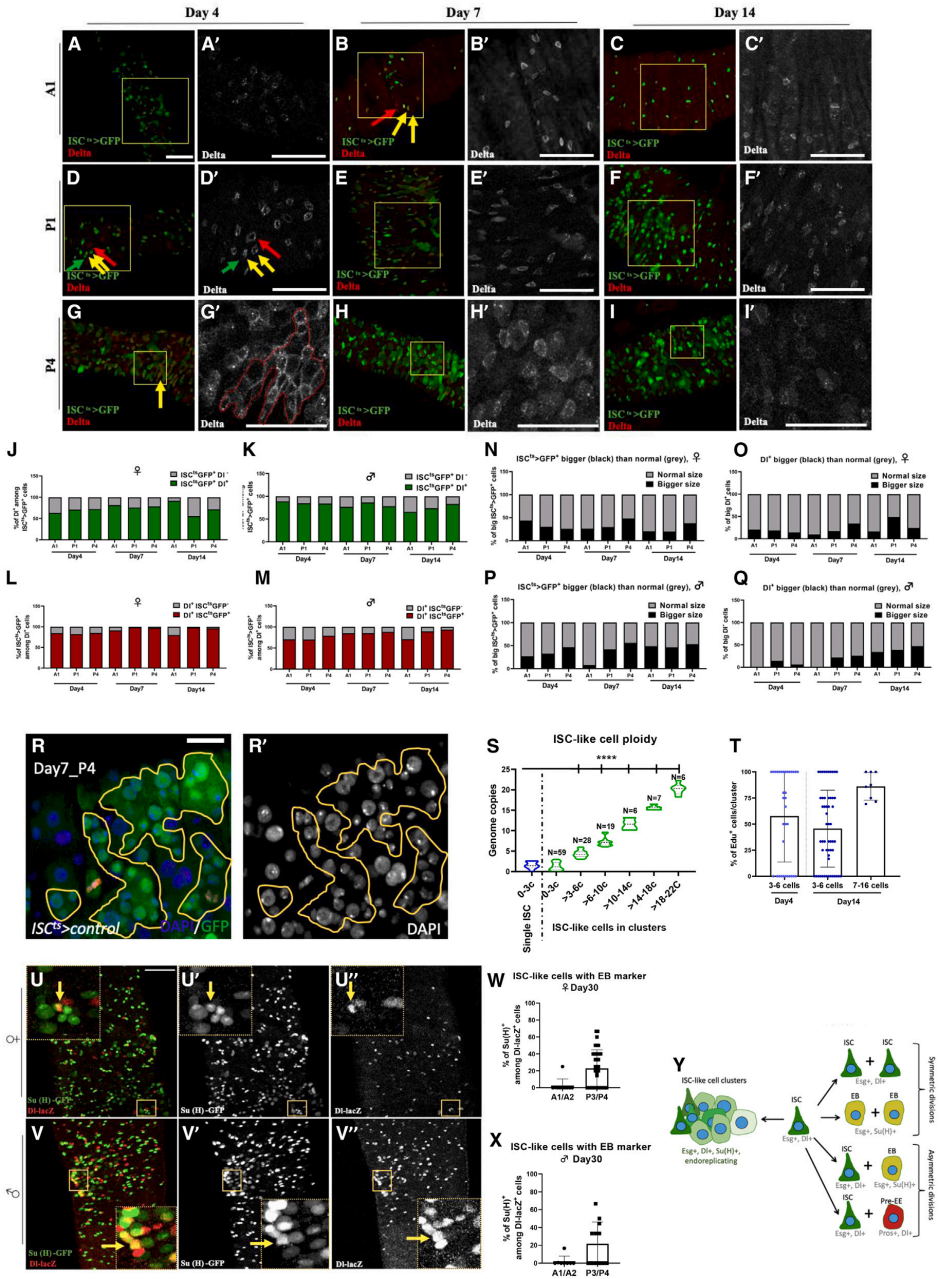
ISC-like cells exhibit ISC^ts^>GFP and Dl expression overlap, occasionally Su(H)-GFP expression and variably increased ploidy and cell size (A–I) *ISC*^*ts*^*>GFP* (green) and Dl*-*stained (red) female midgut regions, A1, P1 and P4, of a *ISC*^*ts*^*-Gal4 UAS-GFP* fly strain outcrossed to *w*^*1118*^ and incubated at 29°C for 4 (A, D, G), 7 (D, E, F) and 14 (G, H, I) days. Red arrows indicate ISCs marked only with Dl, green arrows ISCs marked only with GFP and yellow arrows ISCs marked by Dl and GFP. Frames in A-I indicate regions of Dl staining expanded in A′-I’. (J and K) % of *ISC*^*ts*^*>GFP*-positive cells in females (J) and males (K) that are also Dl-positive (green) or Dl-negative (gray) in A1, P1 and P4 at Day4, 7 and 14 at 29°C (*n* = 4 midguts). (L and M) % of Dl-positive cells in females (L) and males (M) that are also *ISC*^*ts*^*>GFP*-positive (green) or *ISC*^*ts*^*>GFP*-negative (gray) in A1, P1 and P4 at Day4, 7 and 14 at 29°C (*n* = 4 midguts). (N–P) % of *ISC*^*ts*^*>GFP*-positive cells with increased (black) versus normal (gray) surface area in female (N) and male (P) A1, P1 and P4 at Day4, 7 and 14 at 29°C (*n* = 4 midguts). (O-Q) % of Dl-positive cells with increased (black) versus normal (gray) surface area in female (N) and male (P) A1, P1 and P4 at Day4, 7 and 14 at 29°C (*n* = 4 midguts). (R) Outlined ISC-like clusters in the *ISC*^*ts*^*>GFP* female P4 region at day7. Big clusters of ISCs/ISC-like cells (green), stained with DAPI nuclei (blue) (white in R′), were chosen to showcase the extent of increased ploidy. (S) ISC and ISC-like cell DNA content relative quantification using sum projection images of *ISC*^*ts*^*>GFP* female P4 region at day7. The DNA content of each ISC-like cell was divided to that of the average of single ISCs representing 2 DNA copies (2c). One-way ANOVA with correction for multiple comparisons, ∗∗∗∗*p* < 0.0001. (T) Percentage of the EdU^+^ cells found in smaller (3–6 cells) or bigger (7–16 cells) clusters in 4-day and 14-day-old fly midguts. *n* = 34 for 3–6 cells/Day4, *n* = 54 for 3–6 cells/Day14, *n* = 8 for7-16 cells/Day14. (U and V) 30-day-old female and male P3/P4 regions of *Dl-lacZ; Su(H)GBE-nlsGFP* flies. ISCs are marked in red (U′, V′) and EBs in green (U″, V″). Overlap of the two markers (yellow) indicates mixed cell identity. (W and X) Percentage of *Dl-lacZ* positive cells, marked with *Su(H)GBE-nlsGFP* in the P3/P4 region of females (W) and (X) males (*n* ≥ 10 for W and X). (Y) Illustration of anticipated ISCs and progenitors (right) and non-anticipated ISC-like cells (left) as a result of ISC division. Asymmetric division of ISCs can generate an ISC and an EB or an ISC and a preEE, while symmetric ISC divisions predominantly generate two ISCs or two EBs. Each cell type is distinguishable via specific markers. The markers are mixed in cells found within ISC-like clusters. Scale bar: 50 μm for (A–I, U–V), 37.5 μm for (R). See also Figures S2 and S3.

Four-day-old female midguts exhibited clusters of 5–6 Dl-positive cells in the P4 region, whereas 7- and 14-day-old female midguts exhibited clusters of up to 8–20 Dl-positive cells in the P1 and P4 regions (Figures 1Q and 2A–2I). In these relatively young flies Dl+ cell clustering is evident in the posterior midgut and may regionally increase between 4-day-old and older females (Figures 1Q and 2A–2I). Moreover, mosaic analysis with a repressible cell marker (MARCM82B) showed that major clusters of Dl-positive cells in 14-day-old female midguts are formed nearby but not inside clonally produced cells, suggesting that Dl-positive cell clusters are not clonally derived (Figure S1). In conclusion, using three transgene markers as proxies of progenitor cell numbers, as well as Dl staining, we provide evidence that progenitor cell clustering starts early in adult fly life in a regionally biased manner.

### Clustered ISCs have mixed ISC-EB identity and increased ploidy

To assess the specificity of *ISC*^*ts*^*-Gal4 UAS-GFP* expression as a marker of clustered ISCs, we calculated its overlap with Dl protein expression in the A1, P1 and P4 regions of 4-, 7-, and 14-day-old female and male midguts (Figures 2A–2Q). We found that on average ∼75% (55–95%) of GFP-expressing cells in females and ∼80% (70–90%) in males exhibited detectable Dl staining (Figures 2J and 2K). This overlap is superior to the one observed between *Dl-Gal4 UAS-GFP* and Dl protein expressing cells (Figure S2). The percentage of GFP-expressing cells devoid of Dl staining may in theory be ISC-like cells in transit to become EBs, since many of them cluster and are bigger than single GFP-positive cells (Figures 2N and 2P). By dividing the surface area of each of the clustered GFP-positive cell to the mean surface area of single GFP-positive cells from the same image, we calculated the % of clustered GFP-positive cells being bigger than the biggest single GFP-positive cell observed. Remarkably, 14-day-old males had more of those bigger *ISC*^*ts*^*-Gal4 UAS-GFP* cells than 4-day-old males (Figure 2P).

Similarly, on average ∼85% (75–98%) of Dl-positive cells in females and ∼80% (70–95%) in males exhibited detectable *ISC*^*ts*^*-Gal4 UAS-GFP* expression (Figures 2L and 2M). The Dl-positive cells devoid of GFP expression are considered pre-EEs ^5 6 8^. However, some of the Dl-positive cells found in clusters were bigger compared to single Dl-positive cells. (Figures 2O and 2Q). By dividing the surface area of each of the clustered Dl-positive cell to the mean surface area of single Dl-positive cells from the same image, we calculated the % of clustered Dl-positive cells being bigger than the biggest single Dl-positive cell observed. Remarkably, 14-day-old males and females had more of those bigger Dl-positive cells than 4-day-old males and females (Figures 2O and 2Q).

To test if the enlarged *ISC*^*ts*^*-Gal4 UAS-GFP* cells endoreplicate, we stained 7-day-old female midguts with DAPI (Figure 2R) and measured the DNA content of GFP-positive cells in the P4 region, where the ISC-like surface area was increased the most (Figures 2N and 2P). We found that most of the clustered GFP-positive cells were polyploid in reference to the 2c ploidy of single GFP-positive cells (Figure 2S), indicating that *ISC*^*ts*^*-Gal4 UAS-GFP* endoreplicate. Moreover, we assessed DNA replication via EdU (5-ethynyl-2′-deoxyuridine).^56^^,^^57^ We found that ISC-like clusters of 7–16 cells exhibited ∼85% EdU positivity, while clusters of 3–6 cells ∼50% EdU positivity, irrespective of fly age (Figure 2T). In some clusters all cells were EdU-positive (Figure 2T), whereas EdU-positive cells exhibited nucleus size variation in most clusters (Figures S3A and S3B). Thus, ISC-like cells exbibit endoreplication in addition to mitosis, in agreement with the existence of Dl-positive cells that exhibit cell size variations (Figures 2O and 2Q).

To identify if ISC-like cells express markers of other cell types, we used flies of the *Dl-lacZ; Su(H)GBE-Gal4 UAS-GFP* genotype to colocalize Dl with EB marker expression (Figures 2U and 2V). Up to 22% of the *Dl-lacZ*-positive cells expressed the EB marker in posterior hotspot regions of females (Figures 2U and 2W) and males (Figures 2V and 2X). To colocalize ISC-like cells with EE/pre-EE marker expression, the *ISC*^*ts*^*-Gal4 UAS-GFP* flies were stained with EE/pre-EE marker Pros (Figures S3C–S3F). A rather low number of GFP*-*positive cells, 3–4% in females (Figure S3E) and 1–2% in males (Figure S3F), were Pros-positive. To measure the % of cells assuming EC-identity within ISC-like clusters, we crossed flies of the genotype, *Myo1A-Gal4 UAS-GFP; Su(H)-Gal80 tub-Gal80*^*ts*^, to the ISC-specific marker *Dl-lacZ*. We did not detect any EC-marked cells co-expressing *Dl-lacZ* in females (Figure S3G) or males (Figure S3H). Thus, in addition to the typical ISC daughter cells, that is, ISCs, EBs and Pre-EEs, we find clusters of ISC-like cells that express Dl, *esg,* and occasionally Su(H)GBE, and undergo mitosis or endoreplication (Figure 2Y).

### Genetic background boost on young midgut cell mitosis increases ISC-like clustering

**Figure 3:**
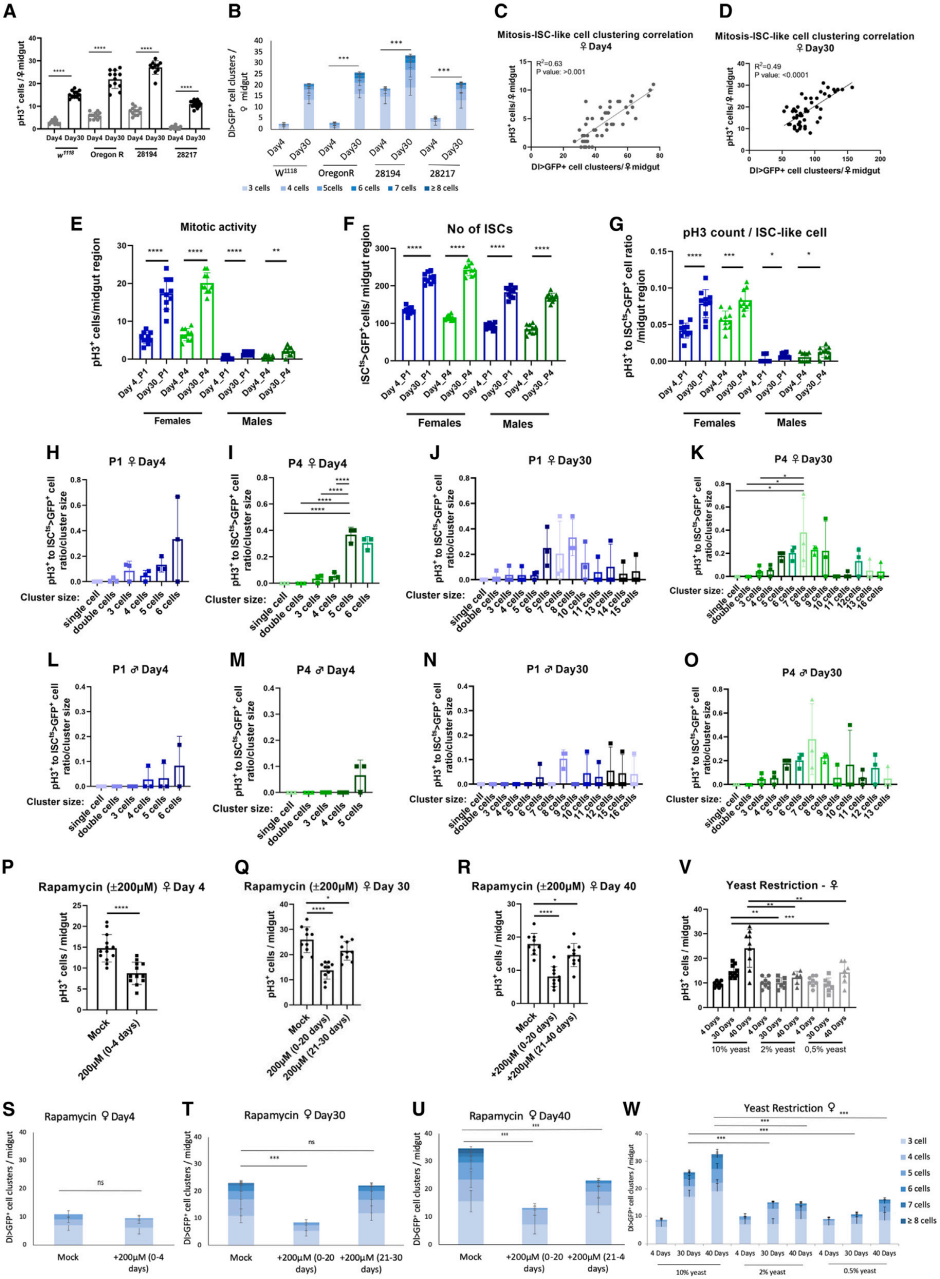
ISC mitosis promotes ISC-like clustering and vice versa (A) Mitotic (pH3^+^) cells per female midgut of flies of a *Dl-Gal4 UAS-GFP* strain outcrossed to *w*^*1118*^ and Oregon-R, or backcrossed to DGRP-28217and DGRP-28194 and incubated for 4-day and 30-day-old flies at 25°C. (B) *Dl>GFP*-positive cell clusters per female midgut of flies and conditions described in A. (C and D) Correlation of the average mitosis rate per female midgut with the corresponding sum of *Dl>GFP*-positive cell clusters of the same midgut; 4 (C) and 30 (D) day old flies of different genetic backgrounds, *w*^*1118*^, Oregon-R, DGRP-28217, DGRP-28194, were pooled together. (E-G) pH3-positive cells (E), *ISC*^*ts*^*>GFP*-positive cells (F) and pH3-positive to *ISC*^*ts*^*>GFP*-positive cell ratio (G) per P1 and P4 midgut regions of 4-day and 30-day-old *ISC*^*ts*^*>GFP* males and females at 25°C. (H-O) Mitosis per ISC/ISC-like cell according to cluster size. pH3-positive to *ISC*^*ts*^*>GFP*-positive cell ratio per cluster size in P1 (H,J,L,N) and P4 (I,K,M,O) region of 4-day-old (H-I, L-M) and 30-day-old (J-K, N-O) female (H-K) and male (L-O) midguts. (P–R) pH3-positive cells per midgut upon administration of 200 μM rapamycin in the fly food to 4- (P), 30- (Q) and 40-day-old (R) female *Dl-Gal4 UAS-GFP* flies outcrossed to Oregon-R, either early in life (0–20 days) or later in life (Day 21–30 for Q and Day 21–40 for R) versus control fly food (mock). (S–U) *Dl>GFP*-positive cell clusters per midgut upon administration of 200 μM rapamycin in the fly food, under the parameters described in P-R. (V) pH3-positive cells per *Dl>GFP* female midgut upon yeast caloric restriction. 10% yeast in food compared to 2% and 0.5% yeast in young (day4) and old (day 30 and day 40) flies. (W) *Dl>GFP*-positive cell clusters per midgut upon yeast caloric restriction, under the parameters described in V. (A, E–G, P, Q, R, V) Student’s *t* test, *n* ≥ 10 midguts (except V, *n* ≥ 8 midguts). (B, S, T, U, W) Chi-square test, expecting equal number of 3+ cell clusters between conditions, *n* = 10 midguts, 1 degree of freedom, ∗∗∗*p* < 0.001, ns, not statistically significant. (C, D) Linear regression (R^2^) and significance assessment using *n* = 49 data points. See also Figure S4.

ISC accumulation in old flies is linked to higher ISC proliferation rate,^26^ because of higher JNK signaling in old flies boosting symmetric ISC divisions producing two ISCs.^34^ However, we noticed ISC-like cell accumulation also in young flies, and we sought to correlate young and old fly mitosis with ISC-like clustering (Figures 3A–3D). *Dl-Gal4 UAS-GFP* flies were crossed at 25°C to *w*^*1118*^, Oregon-R, and two strains of the *Drosophila* Genetic Reference Panel (DGRP) to mark ISCs in four different genetic backgrounds previously shown to exhibit distinct mitotic potential.^52^ The genetic background of the line DGRP-28194 was the most mitotic, followed by that of Oregon-R, while that of the lines DGRP 29217 and *w*^*1118*^ was the least mitotic (Figure 3A).^52^ Enumeration of ISC-like cluster formation in the same midguts, based on GFP expression, showed that young and old flies of the 4 genetic backgrounds exhibited ISC-like clustering roughly corresponding to their level of mitosis (Figures 3A and 3B). For example, more and bigger clusters (of >8 cells) were formed in the young and old midguts of the genetic background of the DGRP-28194 line (Figure 3B). To assess if the mitosis rate correlates with ISC-like clustering in young flies, we performed logistic regression analysis correlating the average mitosis rate in each of the 49 young midgut replicates of all four genotypes pooled together with the corresponding ISC-like cluster measurements. We found that mitosis rate tightly correlated with ISC-clustering in young flies (Figure 3C). There was also a significant correlation between the average mitosis rate in each of the 49 old midgut replicates with their ISC-like cluster measurements (Figure 3D). Thus, high mitosis rate is tightly linked to ISC-like clustering in the young and old flies.

*Drosophila* female ISCs are more proliferative than male ISCs, hence their propensity for age-associated dysplasia.^25^^,^^28^ Accordingly, we found ISC-like clustering in old flies, and increased ISC-like clustering propensity in young females compared to young males (Figures 1A–1F). Regardless of the major sex difference in the mitosis rate, mitotic cells were significantly increased in the P1 and P4 regions of both sexes over time (Figure 3E). Strikingly though, not only the number of ISC-like cells (Figure 3F), but also the mitosis rate per ISC-like cell increased significantly in males and females in the P1 and P4 regions upon aging (Figure 3G). This observation clearly indicates that mitosis rate per ISC-like cell accelerates over time in both sexes.

### ISC-like cells have higher mitotic activity when in clusters of 5 or more cells

Young flies develop clusters of up to 6 cells each, while old flies develop clusters of up to 16 cells each. To assess the mitotic potential of ISC-like cells independently of age and sex, we measured the mitotic rate per ISC-like cell as a function of the number of cells per ISC-like cell cluster in the P1 and P4 regions. To do that we divided the total mitosis to the total number of *ISC*^*ts*^*-Gal4 UAS-GFP* cells within each cluster, while clusters were stratified in the following consecutive categories: single cells (GFP singlets), double cells (GFP duplets), 3 cells (GFP triplets) and so on. We found that the mitotic rate per ISC-like cell increases in clusters of ≥5 cells (Figures 3H–3O). Young females have ISC-like cells in clusters of 5–6 cells exhibiting more mitosis (>20% pH3+ ISCs) than ISC-like cells found in clusters of 1–4 cells (<5% pH3+ ISCs) (Figures 3H and 3I). Similarly, old females had clusters of 1–4 cells exhibiting low mitosis per ISC-like cell (<5% pH3+ ISCs), but they also had midsize clusters of 5–10 cells exhibiting high mitosis (>20% pH3+ ISCs), and clusters of >10 cells, which exhibited lower mitosis per ISC-like cell than midsize cluster cells (<10% pH3+ ISCs) (Figures 3J and 3K). Likewise, young and old males had clusters of 1–4 cells exhibiting very low mitosis per ISC cell, while old males had clusters of 5–16 cells exhibiting tentatively higher mitosis per ISC-like cell, which was more pronounced in midsize clusters (Figures 3L and 3O). We conclude that the mitosis rate per ISC-like cell increases in clusters of 5–10 cells.

### Chemical modulation of mitosis in young flies affects ISC-like clustering during aging

To assess the impact of mitosis on ISC-like cluster formation at various stages of adult life, we treated young and old flies with drugs and diets affecting mitosis. Administering 200 μM rapamycin, a Target of Rapamycin (TOR) kinase inhibitor,^58^^,^^59^ to the young progeny of *ISC*^*ts*^*-Gal4 UAS-GFP* flies outcrossed to *w*^*1118*^ for 4 days reduced mitosis (Figure 3P) without any immediate effect on cluster formation (Figure 3S). However, feeding 200 μM rapamycin for 20 days upon eclosion reduced mitosis (Figures 3Q and 3R) and cluster formation (Figures 3T and 3U) in 30-day-old and 40-day-old flies. The effect was less pronounced when rapamycin was administered later in life (Figure 3Q–3R, 3T, and 3U). We reproduced these results using the progeny of *ISC*^*ts*^*-Gal4 UAS-GFP* flies outcrossed to Oregon-R (Figures S4A–S4F). Thus, rapamycin administration early in adult life significantly reduces mitosis and ISC-like clustering in old flies.

Similarly, administering 200 μM Floxuridine, an inhibitor of DNA and RNA synthesis,^60^ for 4 days following eclosion (young flies) tentatively reduced mitosis (Figure S4G) without affecting cluster formation (Figure S4J). However, it reduced mitosis and cluster formation significantly in 30-day-old and 40-day-old flies, and at least as affectively as Floxuridine administration during the last 10 days of fly life (Figures S4H, S4K, S4I, and S4L). Thus, mitosis reduction in young flies reduces ISC-like cell clustering later in life.

On the other hand, dietary restriction of *ISC*^*ts*^*-Gal4 UAS-GFP* flies outcrossed to Oregon-R (Figures 3V and 3W) or *w*^*1118*^ (Figures S4M and S4N) via feeding on 2% or 0.5% yeast instead of 10% yeast, reduced mitosis and cluster induction in old flies, without affecting mitosis or clustering at 4 days (Figures 3V, 3W, S4M, and S4N). Thus, unlike chemotherapy drugs that can reduce mitosis before reducing ISC-like cell clustering, dietary yeast restriction jointly reduces mitosis and ISC-like cell clustering only later in life.

### Reduced chromatin accessibility of *Notch* pathway genes and *Notch* locus position-effect variegation in young progenitors predisposes for ISC-like cell accumulation

ISCs divide at baseline levels in young homeostatic midguts, while in aged midguts, various mitogens contribute to ISC overproliferation.^31^^,^^32^^,^^61^ However, the reason of ISC progeny differentiation loss during aging remains unclear. Given that spontaneous tumors and Notch signaling deregulation is evident late in life,^36^ while ISC-like cell clustering starts with high penetrance in the posterior midgut early in life, we hypothesized that young posterior midgut progenitors might exhibit reduced chromatin accessibility or epigenetic instability affecting Notch pathway controlling loci.

**Figure 4:**
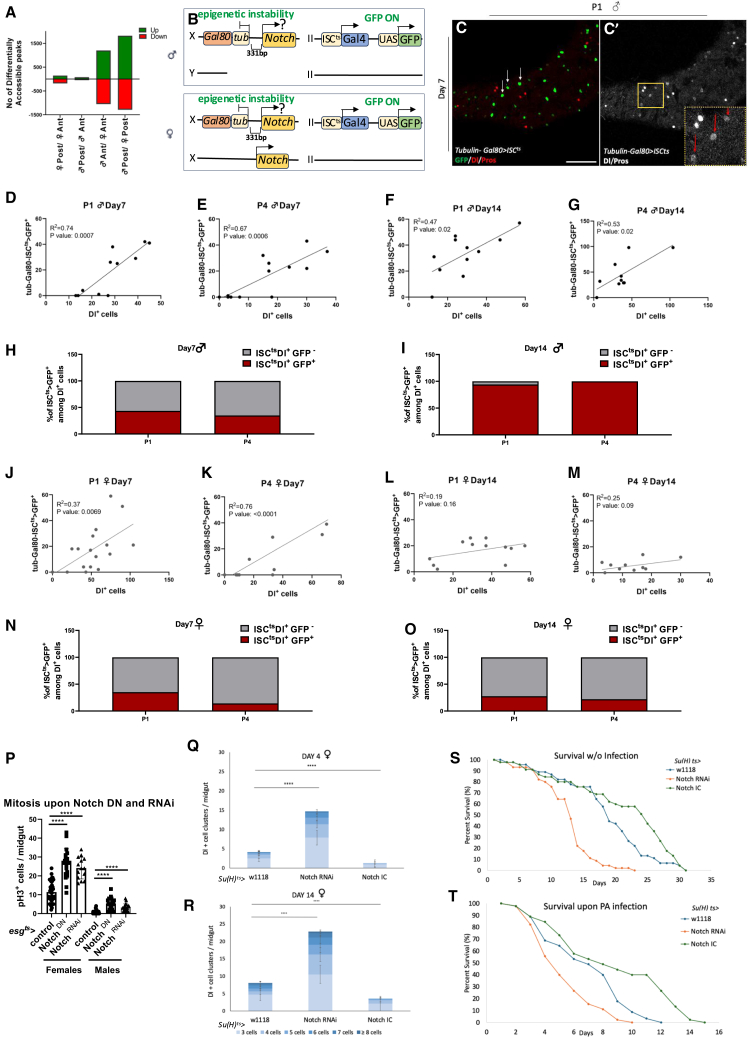
ATAC-seq and analysis via the *NotchTSS-331tubGal80* position-effect variegation tool (A) Number of differential chromatin accessibility peaks being higher (green) or lower (red) in the posterior versus anterior midgut of females or males, and peaks being higher (green) or lower (red) in males versus females regarding anterior or posterior midgut. Peaks were included at *p*-value <0.05. (B) Illustration of *NotchTSS-331tubGal80* position-effect variegation tool in males and females used in the analysis: endogenous *Notch* gene, and the *tubulin-Gal80* transgene integrated in the opposite orientation 331 bp upstream of the Notch transcription starting site, on X chromosome, crossed to *esg*^*ts*^*-Gal4 UAS-GFP* (located on chromosome II) or *ISC*^*ts*^*-Gal4 UAS-GFP* (not shown). Females contain an extra, untagged copy of Notch. (C) P1 region of a *NotchTSS-331tubGal80; ISC*^*ts*^*-Gal4, UAS-GFP* young male incubated for 7-day at 29°C. Notch locus silencing induces GFP expression (green). (C′) ISCs are marked by Dl (spotty red) and EEs and pre-EEs by pros (solid red). ISCs expressing GFP (red arrows). (D–G, J–M) Correlation between the number of GFP^+^ cells in *NotchTSS-331tubGal80 ISC*^*ts*^*-Gal4/UAS-GFP* young males and females and Dl^+^ cells in P1 and P4. Positive correlation between GFP^+^ cells and Dl^+^ cells in males (D–G) and females (J–M) on day 7 (D-E) and 14 (F-G) post induction. Linear regression (R^2^) and significance assessment using *n* ≥ 10 data points. (H, I, N, O) % of Dl-positive cells in *NotchTSS-331tubGal80 ISC*^*ts*^*-Gal4/UAS-GFP* males (H,I) and females (N,O) that express (red) or do not express (gray) in P1 and P4 at Day7 (H,N) and Day14 (I,O) at 29°C (*n* = 5 midguts). (P) pH3-positive cells per midgut upon Notch^RNAi^ or Notch^DN^ expression via *esg*^*ts*^*-Gal4* in females and males incubated for 3 days at 29°C. Student’s *t* test, *n* ≥ 15 midguts. (Q and R) Dl-positive cell cluster per female midgut expressing *Notch*^*RNAi*^ or activated *Notch (Notch*^*IC*^*)* in EBs using *Su(H)GBE*^*ts*^*-Gal4* for 4 days (U) and 14 days (V) at 29°C. Chi-square test, expecting equal number of 3+ cell clusters between conditions, *n* = 10 midguts, 1° freedom, ∗∗∗∗*p* < 0.0001. (S and T) Survival at 29°C of *Notch*^*RNAi*^, *Notch*^*IC*^ and control *w*^*1118*^ lines outcrossed to *Su(H)GBE*^*ts*^*-Gal4* upon intestinal infection (S) or rearing in standard cornmeal (T). *n* = 50 flies per condition. Scale bar: 50 μm for (C). See also Figures S5–S9.

To explore the epigenetic profile of ISC-like cells, we performed ATAC-sequencing of FACS-sorted young anterior and posterior, *esg*^*ts*^*-Gal4 UAS-GFP* midgut progenitors. We found more anteroposterior differences in chromatin accessibility in female compared to male progenitors, and even more differences between males and females in the anterior and the posterior midgut regions (Figure 4A). The more differences between females and males in chromatin accessibility mirror the higher mitosis and ISC-like cell clustering in the posterior versus the anterior midgut of females, and the overall low mitotic activity in the anterior and the posterior midgut of males.

Performing KEGG pathway analysis of differentially accessible (DA) genes in anterior versus posterior female midgut progenitors we primarily found DA peaks in core members of the Notch and TGF-beta pathways (Figure S5A). For example, each of the Notch pathway genes, *Notch, mastermind* (*mam*) and *numb,* contained one peak of lesser accessibility in the posterior compared to anterior female progenitors, and less overall accessibility in females compared to males (Figures S5B–S5D). This agrees with the ∼33% reduced expression of *Notch* in the posterior versus the anterior female midgut EBs^19^ and suggests that there is plasticity in the expression of genes that affect *Notch* signaling in a regional manner.

Moreover, we noticed that Notch protein expression among midgut epithelial cells is not even. Antibody staining against the extracellular domain of Notch showed specific extranuclear signal in essentially all *esg*^*ts*^*-Gal4 UAS-GFP* positive cells (Figures S6A and S6B). However, these cells exhibited cell to cell difference in the intensity of Notch expression (Figure S6C). Accordingly, we sought to monitor *Notch* locus epigenetic instability in the midgut progenitor cells, by developing *NotchTSS-331tubGal80,* a position-effect variegation detection tool.^62^ By inserting a *tubGal80* transgene cassette 331 bp upstream of the *Notch* transcription start site (TSS) and by coupling it with the *ISC*^*ts*^*-Gal4 UAS-GFP*, we measured the percentile of progenitor cells with unscheduled GFP expression out of all Dl+ cells as a proxy of *Notch* locus instability, and correlated the number of GFP+ cells with the number of Dl-positive cells in the P1 and P4 midgut regions. We found that males exhibited a higher percentile of GFP+ to Dl+ cells (35–100%) compared to females (15–35%) at 7- and 14-day-old flies incubated at 29°C (Figures 4H, 4I, 4N, and 4O), and co-abundance of GFP+ with Dl+ cells was stronger in 14-day-old males than females of the same age (Figures 4F, 4G, 4L, and 4M), likely because *Notch* expression instability on the one marked chromosome of females can be rescued by *Notch* expression on the homologous X chromosome (Figure 4B). Finally, co-abundance of GFP+ with Pros+ cells in males or females was broadly insignificant (Figures S7E–S7L).

To verify that the genomic insertion of *NotchTSS-331tubGal80* transgene cassette by itself does not interfere with progenitor cell activity, we introgressed it via backcrossing for ten generations into an *Act5c-Gal4 UAS-GFP* genetic background and then crossed the introgressed insertion (and its isogenic control) to the *ISC*^*ts*^*-Gal4 UAS-GFP* line. Along with sporadic expression of GFP in midgut progenitors, we found a comparable number of mitotic cells (Figure S7B), Dl-positive cells (Figure S7C) and Pros-positive cells (Figure S7D) in the posterior midgut of isogenized, *NotchTSS-331tubGal80 ISC*^*ts*^*-Gal4 UAS-GFP,* versus control, *ISC*^*ts*^*-Gal4 UAS-GFP,* males confirming that the *NotchTSS-331tubGal80* insertion does not impact progenitor cell mitosis or differentiation. Moreover, we found no evidence of sporadic genomic loss the *tubGal80* insertion in males because: (a) Notch protein expression, regardless of its variability in intensity, was detectable in essentially all *ISC*^*ts*^*-Gal4* and *esg*^*ts*^*-Gal4,* GFP positive cells (Figures S8A and S8B), and (b) GFP de-repression in ISCs and EBs was very frequent, and cells exhibited size variation (Figures S8A and S8B), which is unlike the rare and uniform ISC tumor cells that accompany the spontaneous loss of Notch signaling in old males.^36^ Notwithstanding Notch protein turnover that may differ from that indicated by *ISC*^*ts*^*-Gal4 UAS-GFP* expression when using the *NotchTSS-331tubGal80* transgene cassette, higher *Notch* locus epigenetic instability may result in more ISC-like cells in young flies, similarly to *Notch* downregulation in young progenitor cells, which exhibit increased mitosis (Figure 4P) and expands the ISC population.

To assess if the naturally unstable or inefficient *Notch* signaling between ISCs and EBs impacts ISC-like clustering, we expressed *Notch*^*RNAi*^ and activated *Notch* (*Notch*^*IC*^) in the EBs of 4- and 14-day-old females using the *Su(H)*^*ts*^*-Gal4* [*Su(H)GBE-Gal4 UAS-CD8GFP tub-Gal80*^*ts20*^]. We found that *Notch*^*IC*^ expression in EBs reduced ISC-like clustering in very young (Figure 4Q) and older (Figure 4R) flies, secured their commitment toward the EC fate (Figures S9A–S9D), and increased overall survival and survival to infection (Figures 4S and 4T). This suggests that Notch signaling in the posterior midgut EBs is suboptimal and that *Notch*^*IC*^ expression can reinforce ISC progeny differentiation and homeostasis.

### Young ISC-like cells exhibit increased DNA replication stress while clustering

**Figure 5:**
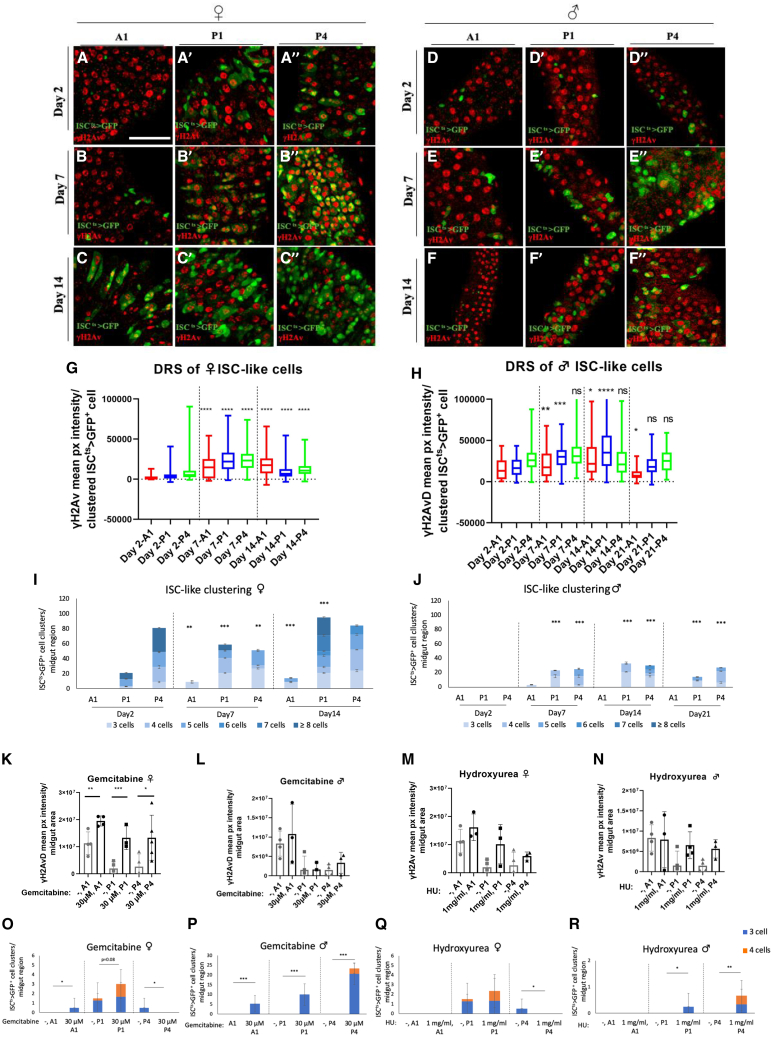
Ubiquitous early onset γH2Av expression in ISCs indicates DRS and ISC-like clustering (A-F) γH2Av expression in the A1 (A-F), P1 (A′-F′) and P4 (A″-F″) regions of 2- (A, D), 7- (B, E) and 14-day-old (C, F) *ISC*^*ts*^*-GFP* females (A-C) and males (D-F). (G and H) γH2Av mean pixel intensity per clustered *ISC*^*ts*^*>GFP*-positive cell in the A1, P1 and P4 of 2-, 7- and 14-day-old females (G) and 2-, 7-, 14- and 21-day-old males (H). Student’s *t* test, *n* ≥ 6 midguts. (I and J) *ISC*^*ts*^*>GFP*-positive cell clusters in the A1, P1, and P4 of 2-, 7- and 14-day-old females (I) and 2-, 7-, 14- and 21-day-old males (J). Chi-square test, n = 3–4 midguts. (G-J) Significance compared to the corresponding region of 2-day-old midguts. (K–N) γH2Av mean pixel intensity per A1, P1 and P4 midgut area of young *ISC*^*ts*^*>GFP* females (K, M) and males (L,N) upon 2 days’ feeding on fly food containing 30μM gemcitabine (K, L) or 1 mg/mL HU (M,N) versus normal food. One-way ANOVA, n = 3–5 midguts. (O–R) *ISC*^*ts*^*>GFP*-positive cell clusters corresponding to the conditions and flies analyzed in (K-N), respectively. Chi-square test, n = 3–4 midguts. Scale bar: 75 μm for (A-F). ∗*p* < 0.05, ∗∗*p* < 0.01, ∗∗∗*p* < 0.001, and ∗∗∗∗*p* < 0.0001.; ns, not statistically significant. See also Figure S10.

DDR, reflected by nuclear γH2AΧ foci, occurs in aging cells due to accumulating DSBs.^63^^,^^64^^,^^65^ Importantly, γH2AΧ can also be induced in response to increased DRS in the absence of DSBs.^43^^,^^66^ Accordingly, a widespread increase in the γH2AvD or ATM/ATR signal in young *Drosophila* midgut ISCs would indicate DRS, while sporadic nuclear foci of the same markers would indicate DDR. We measured the intensity of γH2AvD staining in ISC-like cells in 2-, 7-, 14-, and 21-day-old *ISC*^*ts*^*-Gal4 UAS-GFP* females and males (Figures 5A–5F). We observed significant widespread increases in γH2AvD intensity in ISC-like cells past day 2 in all regions tested, A1, P1 and P4 (Figures 5G and 5H). Similarly, ATM/ATR staining intensity in ISC-like cells was widespread and increased significantly in 14- and 21-day-old compared to 4-day-old flies (Figures S10A–S10G and S10J).

Moreover, we quantified the γH2AvD and ATM/ATR signal over time collectively in all midgut cells (rather than specifically in ISC-like cells) of the A1, P1 and P4 regions. In this case, the increase in staining intensity was tentative (Figures S10I, S10L, S10M, and S10N). Notably, in this analysis most γH2AvD and ATM/ATR signal came from ECs that dominate the midgut area. We conclude that, DRS is widespread during aging and increases statistically in DNA replicating ISC-like cells, and possibly in maturing ECs.

To assess whether ISC-like cells exhibit increased DRS while clustering, we quantified the number of ISC-like clusters comparing them to DRS signal patterns in the same midguts. We found that increased DRS signal past the initial time point (Figures 5G, 5H, S10G, and S10J) coincided with increased ISC-like clustering (Figures 5I, 5J, S10H, and S10K) along the 2 to 21 days’ time course.

Since the γH2AvD signal was widespread and increased over time (Figures 5A–5J and S10), indicative of DRS rather than DDR, we fed flies with two chemical inducers of DRS, gemcitabine and hydroxyurea (HU) and assessed γH2AvD signal intensity and ISC-like cell clustering. Gemcitabine is a nucleoside analog that incorporates in the DNA and induces DRS by inhibiting the synthesis of deoxyribonucleotides.^67^ Similarly, HU inhibits deoxyribonucleotide synthesis and slows down S-phase progression.^68^ We fed young (2-day-old) *ISC*^*ts*^*-Gal4 UAS-GFP* females and males with 30 μM gemcitabine for 2 days at 29°C. The level of γH2Av signal was significantly higher upon gemcitabine feeding in females (Figure 5K) and tentatively higher in males (Figure 5L). Similarly, the γH2Av signal was tentatively higher upon 2 days of 1 mg/ml HU administration in young (2-day-old) flies of both sexes (Figures 5M and 5N) suggesting that the two markers detected DRS in young flies. Importantly, DRS drug administration increased ISC-like cluster formation in the P1 and P4 midgut regions (Figures 5O–5R), indicating the DRS can induce γH2Av and ISC-like clustering.

### *His2Av* or *spn-B* silencing inhibits DRS and ISC-like clustering

**Figure 6:**
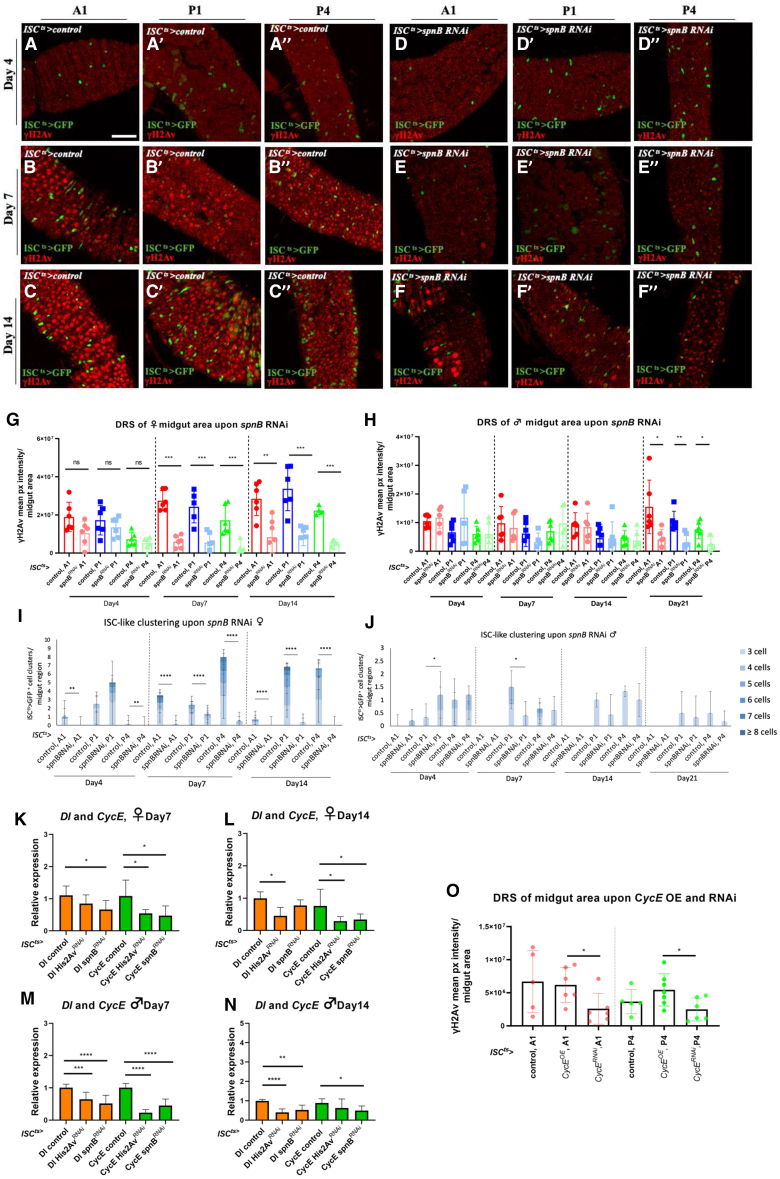
*spn-B* accelerates ISC-like clustering during aging (A-F) γH2Av expression in the A1 (A-F), P1 (A′-F′) and P4 (A″-F″) regions of young ISCts-Gal4 UAS-GFP (A-C) and ISCts-Gal4 UAS-GFP, UAS-spn-BRNAi (D-F) females induced for 4- (A, D), 7- (B-E) and 14-day (C, F) at 29°C. (G and H) γH2Av mean pixel intensity per A1, P1 and P4 midgut area of young ISCts-Gal4 UAS-GFP versus ISCts-Gal4 UAS-GFP, UAS-spn-BRNAi females induced for 4-, 7- and 14-day (G), and males induced for 4-, 7-, 14- and 21-days (H) at 29°C. One-way ANOVA, *n* = 6 midguts. (I and J) ISCts>GFP-positive cell clusters corresponding to the same flies described in G and H, respectively. Chi-square test, *n* = 6 midguts. (K–N) Relative expression of Dl and CycE in ISCts-Gal4 UAS-GFP female (K-L) and male (M-N) midguts co-expressing UAS-His2AvRNAi or UAS-spn-BRNAi for 7 (K, M) or 14 days (L, N). Mann-Whitney U test *n* ≥ 6 biological replicates. (O) γH2Av mean pixel intensity per A1 and P4 region upon ISC-specific knockdown or overexpression of CycE. Mann-Whitney U test, n = 4–7 (females and males in equal parts). Scale bar: 50 μm for (A-F). ∗*p* < 0.05, ∗∗*p* < 0.01, ∗∗∗*p* < 0.001, and ∗∗∗∗*p* < 0.0001.; ns, not statistically significant. See also Figure S11.

To examine the role of DRS in ISC-like clustering, we knocked down *Histone H2A variant (His2Av)* and the DRS-related paralog of *rad51*, *spn-B*^69^ in ISCs using *ISC*^*ts*^*-Gal4* and measured the γH2Av signal. Female midguts exhibited a significant reduction of the γH2Av signal at 7-, 14- and 21-days of *UAS-spn-B*^*RNAi*^ (Figures 6A–6G) or *UAS-His2Av*^*RNAi*^ (Figures S11A–S11G) expression at 29°C. This reduction was coupled with a significant decrease of ISC-like clusters (Figures 6I and S11I), most prominently upon *spn-B* knockdown, in which case clusters were >3-fold less compared to the wild-type control, and no ISC-like cluster was larger than 5 cells (Figure 6I). ISC-specific silencing of *spn-B* or *His2Av* in males reduced the γH2Av signal and ISC-like clustering at the 21-day timepoint (Figures 6H–6J, S11H, and S11J). We conclude that DRS contributes to the expansion of ISC-like clusters.

To further understand how DRS facilitates ISC-like clustering, we downregulated *His2Av* and *spn-B* in ISCs for 7 and 14 days at 29°C and measured the expression of cell cycle regulators, mitogens and ligands of the Notch, Jak/Stat, EGFR, JNK, Toll, and Insulin pathways in whole midguts of adult flies. We found no reduction in the expression of a panel of ligand mitogens (Figures S11K–S11N), most of which are expressed in EBs and developing ECs in response to EC stress or loss. However, *Dl* and the cell cycle regulator *Cyclin E (CycE)*, two genes specifically expressed in progenitor cells, were significantly reduced upon *His2Av* and/or *spn-B* silencing (Figures 6K–6N). While Dl may promote mitosis by establishing and preserving the stemness of ISCs, CycE induces mitosis directly.^70^ To assess the role of CycE we modulated its expression via ISC^ts^-Gal4 measuring the γH2Av signal in the A1 and P4 regions of young females and males. CycE overexpression increased γH2Av signal compared to CycE silencing in both regions at 7 and 14 days (Figure 6O). We conclude that DRS triggers Dl and CycE expression and vice versa promoting ISC-like clustering in relatively young flies.

### Aging-related mitogens induce γH2Av and ISC-like clustering in young and old flies

**Figure 7:**
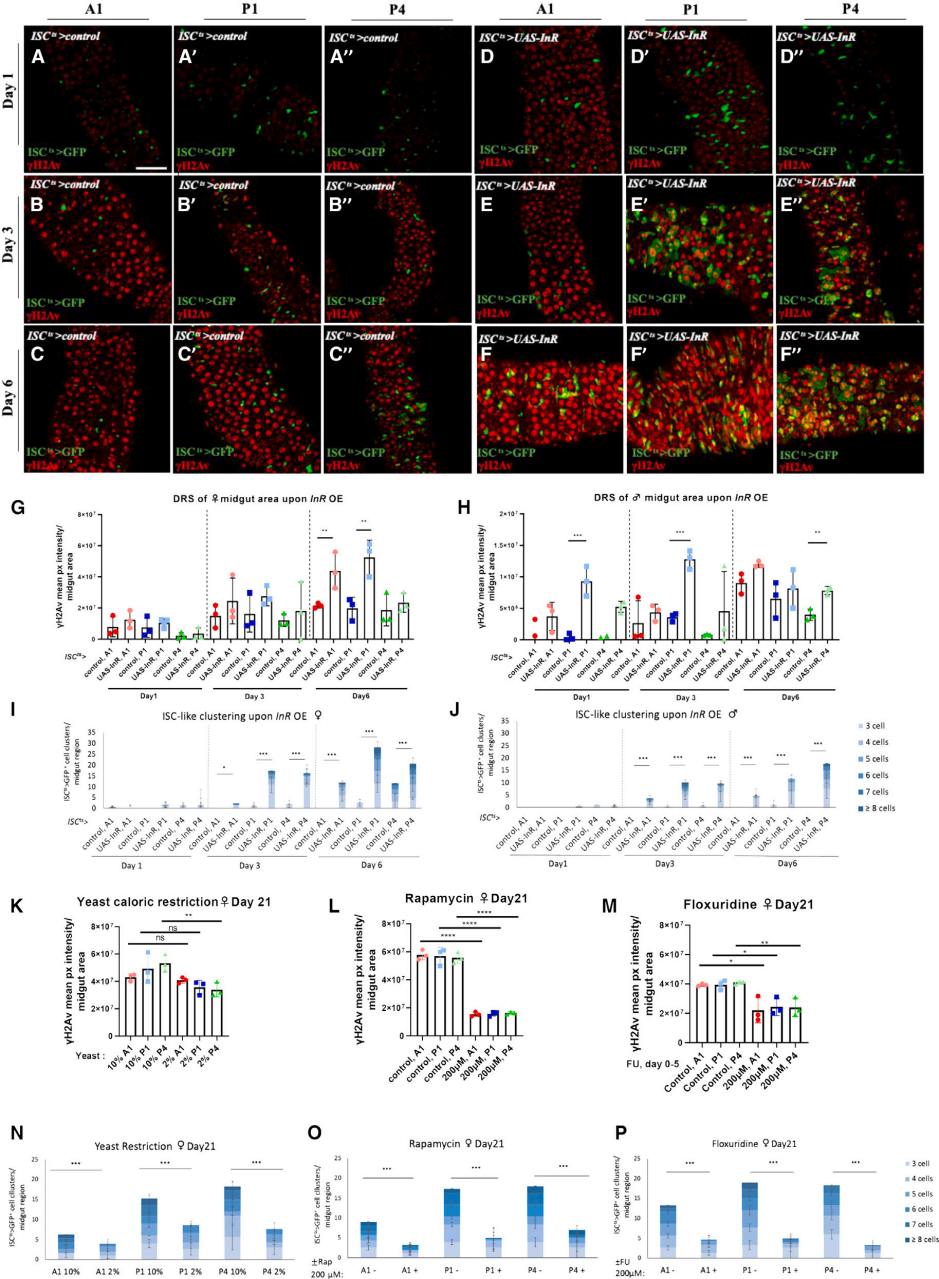
InR increases while anti-mitogenic drugs and yeast caloric restriction reduce DRS and ISC-like clustering (A-F) γH2Av expression in the A1 (A-F), P1 (A′-F′) and P4 (A″-F″) regions of young *ISC*^*ts*^*-Gal4 UAS-GFP* (A-C) and *ISC*^*ts*^*-Gal4 UAS-GFP, UAS-InR* (D-F) females induced for 1- (A, D), 3- (B-E) and 6-day (C, F) at 29°C. (G-H) γH2Av mean pixel intensity per A1, P1 and P4 midgut area of young *ISC*^*ts*^*-Gal4 UAS-GFP* versus *ISC*^*ts*^*-Gal4 UAS-GFP, UAS-InR* females (G) and males (H) induced for 1-, 3- and 6-day at 29°C. One-way ANOVA, *n* = 3 midguts. (I and J) *ISC*^*ts*^*>GFP*-positive cell clusters corresponding to the same flies described in G and H, respectively. Chi-square test, *n* = 6 midguts. (K–M) γH2Av mean pixel intensity per A1, P1 and P4 midgut area of young *ISC*^*ts*^*-Gal4 UAS-GFP* females feeding at 29°C for 21 days on fly food containing 10% or 2% yeast (K), 200 μM Rapamycin or control food (L), 200 μM Floxuridine or control food (M). One-way ANOVA, *n* = 3 midguts. (N–P) *ISC*^*ts*^*>GFP*-positive cell clusters corresponding to conditions and flies described in K, L and M, respectively. Chi-square test, *n* = 6 midguts. Scale bar: 50 μm for (A–F). ∗*p* < 0.05, ∗∗*p* < 0.01, ∗∗∗*p* < 0.001, and ∗∗∗∗*p* < 0.0001.; ns, not statistically significant. See also Figure S12.

To assess the role of ISC mitosis using mitogens relevant to the aging process, we overexpressed the insulin receptor (InR) in ISCs of young female and male midguts via *UAS-InR* for 1-, 3- and 6-day at 29°C (Figures 7A–7F), and quantified γH2AvD intensity (Figures 7G and 7H) as well as the number of clusters in A1, P1 and P4 (Figures 7I and 7J). In females, ISC-like clusters were significantly increased between day 1 and day 3 and more so on day 6, alongside with the γH2Av signal increase (Figures 7G and 7I). Importantly, InR overexpression induced γH2Av (Figure 7G). In males, DRS was measurable even on day 1 upon InR overexpression, and ISC-like clustering followed at the next time points (Figures 7H and 7J).

Furthermore, we induced the expression of the JNK kinase, Hemipterus (Hep), in ISCs via *UAS-hep*^*wt*^ for 1-day, 3-day and 6-day at 29°C (Figure 7S), and we quantified γH2AvD intensity (Figures S12A and S12B) as well as the number of ISC-like clusters in A1, P1 and P4 (Figures S12C and S12D). In females, ISC-like clusters were increased between day 1 and day 3, alongside the increase in γH2Av signal (Figures S12A and S12B). In males, DRS was increased on day 1 upon InR overexpression, and an increase in ISC-like clustering followed at the next time points (Figures S12B and S12D).

Moreover, we reduced mitosis via dietary and pharmacological means. Reducing yeast concentration in the food, from 10% to 2% reduced γH2Av signal in the P4 and tentatively so in the P1 region in 21-day-old midguts at 29°C (Figures 7L and 7M). Administering 200μM Rapamycin in the fly food for 21 days at 29°C, or 200μM of Floxuridine for the first 5 days of fly life reduced and γH2Av signal (Figures 7K–7M) and ISC-cell like clustering in 21-day-old midguts (Figures 7N–7P). Therefore, disparate aging-related mitogens can induce DRS and ISC-like clustering.

### ISC-like clustering in young and old flies correlates with old age mutation-driven tumors

**Figure 8:**
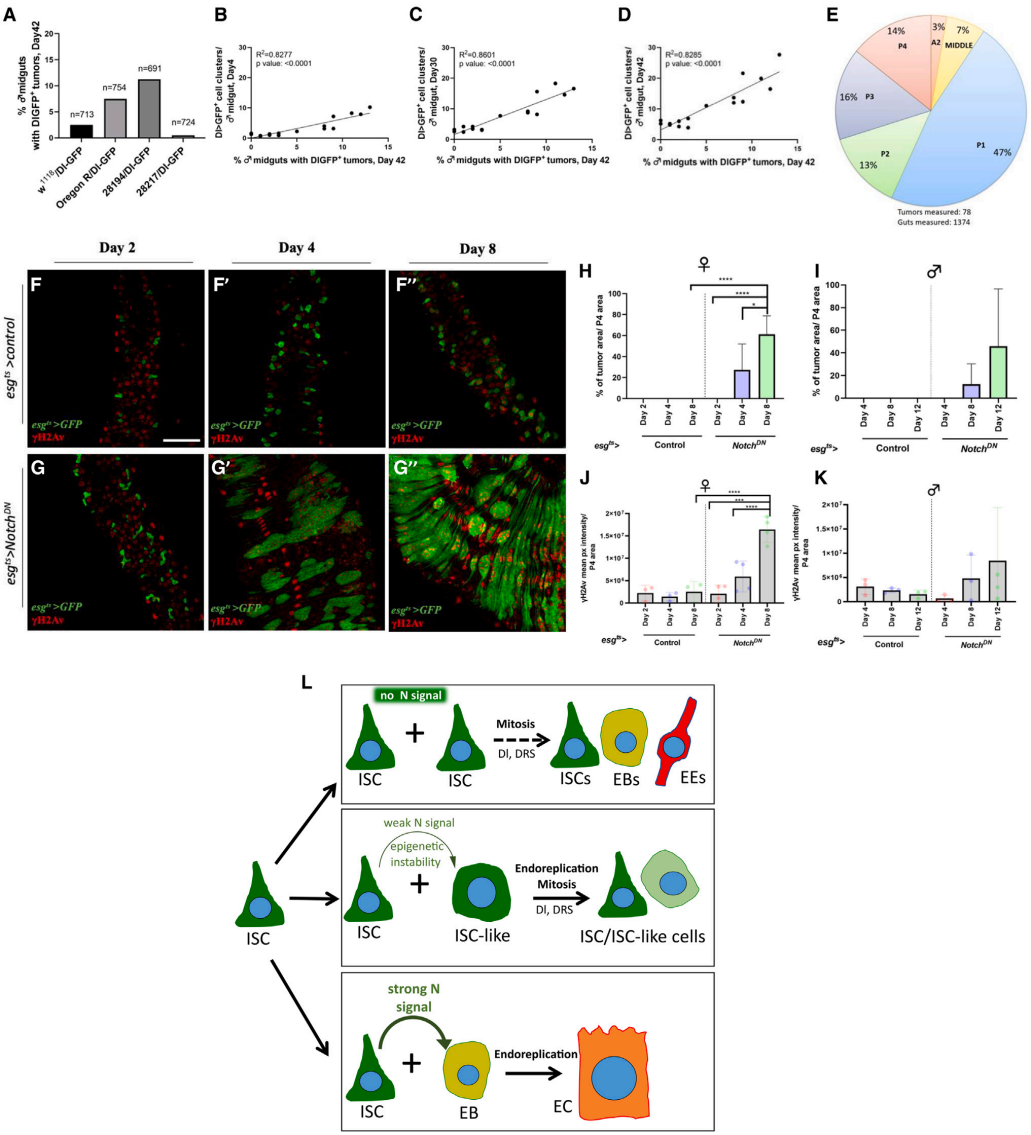
Early onset ISC-like cell clusters are linked to late life spontaneous tumor formation and DRS (A) % of male flies raised for 42 days at 25°C that develop *Dl>GFP*-positive tumors in their midguts. Flies used were the total progeny of *Dl-Gal4 UAS-GFP* flies outcrossed to *w*^*1118*^ (*n* = 713) and Oregon-R (*n* = 754), backcrossed to DGRP-28194 (*n* = 691) and DGRP-28217 (*n* = 724) performed in 4 replicate experiments. (B-D) Pearson correlation of total *Dl>GFP*-positive cell clusters per male midgut at 4- (B), 30- (C), and 42-day (D) at 25°C with the % of male flies raised for 42 days at 25°C that develop *Dl>GFP*-positive tumors in their midguts. *n* = 16 data points were used for each correlation, based on 4 experiments per each of the 4 genotypes described in A. Linear regression (R^2^) and significance assessment using *n* = 16 data points. (E) % of the *n* = 78 *Dl>GFP*-positive tumors identified in distinct male midgut regions by screening 1374 progeny raised for 42 days at 25°C of flies of the 4 genotypes described in A. (F and G) γH2Av mean pixel intensity per P4 midgut region (red) and ISCs and progenitors (green) of *esg*^*ts*^*-Gal4 UAS-GFP* (F) and *esg*^*ts*^*-Gal4 UAS-GFP UAS-Notch*^*DN*^ (G) females induced for 2- (F-G), 4- (F′-G′) and 8-day (F″-G″) at 29°C. Scale bar: 50 μm (H and I) % of females raised at 29°C for 2-, 4- and 8-day (H) and males raised at 29°C for 4-, 8- and 12-day (I) that develop *esg*^*ts*^*>GFP*-positive tumors in the P4 region upon *UAS-Notch*^*DN*^ expression compared to control. (J and K) γH2Av mean pixel intensity per P4 midgut region of *esg*^*ts*^*-Gal4 UAS-GFP* and *esg*^*ts*^*-Gal4 UAS-GFP UAS-Notch*^*DN*^ females (J) and males (K). One-way ANOVA, *n* = 3 midguts. ∗*p* < 0.05, ∗∗*p* < 0.01, ∗∗∗*p* < 0.001, and ∗∗∗∗*p* < 0.0001. (L) Three scenarios on the fate of ISC progeny according to the strength of Notch signaling reception: Genetically intact ISCs signal prospective EBs via Dl/Notch. If Notch signal reception is unidirectionally strong, an ISC and an EB are formed. The latter differentiates to become an EC. If Notch signal reception is weak due to epigenetic instability, an ISC and an EB are formed, but the latter lingers in an ISC-EB mixed identity state producing ISC/ISC-like cells. Finally, if Notch signal reception is essentially absent, e.g., due to high Dpp/BMP signaling, two ISCs are formed.

DNA damage in ISCs and mutation-driven tumors have been described in old flies.^36^^,^^37^ These tumors are rare, and contain uniform ISCs and EEs in numbers up to 100 times higher compared to cells found in ISC-like clusters. This is because tumors are driven by mutations, predominantly those in Notch pathway genes.^36^ To assess the role of ISC-like clustering in tumor formation upon aging, we outcrossed *Dl-Gal4 UAS-GFP* to *w*^*1118*^, Oregon R, and DGRP lines 28194 and 28217, enumerating ISC-like cells at 4, 30 and 42 days and tumors at 42 days (Figure 8A). Strikingly, the level of ISC-like clustering at 4, 30 or 42 days correlated significantly with tumor formation at 42 days (Figures 8B–8D). Moreover, 90% of tumors appeared in the posterior midgut (47% in P1, 13% in P2, 16% in P3, 14% in P4, 7% in middle and 3% in A2 region) (Figure 8E), in agreement with previous work.^36^ Our results suggest that ISC-like cells precede and may predispose for mutation-driven midgut tumors in old adults.

### Notch downregulation creates tumors that express high levels of γH2Av

To assess DRS within *Notch* loss of function-driven tumors, we expressed a dominant negative form of *Notch* (*UAS-Notch*^*DN*^) in ISCs and EBs using *esg*^*ts*^*-Gal4* in females for 2, 4 and 8 days and males for 4, 8, 12 days at 29°C. Tumors started to develop by day 4 and became very prominent by day 8 in females (Figures 8F–8H), while in males, tumors were first apparent by day 8 and very prominent by day 12 (Figure 8J). Importantly, γH2Av intensity was induced over time following the timing of tumor formation (Figures 8H and 8I and 8J-K). This suggests that ISC/ISC-like clustering enhances DRS presumably due to higher replication rate of tumor cells and the paracrine mitogens they emit.

## Discussion

*Drosophila* midgut ISCs serve as the cells of origin of genetic or old age mutation-driven tumors.^16^^,^^51^^,^^71^^,^^72^ Spontaneous mutation of *Notch* pathway genes in old flies creates large tumors of uniform ISCs and EEs.^10^^,^^16^^,^^36^ But when and how ISCs start malfunctioning during spontaneous aging? Here, we find that dysplasia starts sporadically in young flies due to an inherently flawed signaling between ISCs and EBs, which produces clusters of molecularly and cellularly heterogeneous ISC-like cells. Unlike reports focusing on spontaneous tumors and dysplasia in old flies, we find ISC-like cell clusters in essentially all male and female midguts, starting at a very young age. For that we used 4 markers, Dl staining, *Dl-Gal4 UAS-GFP*, *Dl-lacZ*, and *ISC*^*ts*^*-GAL4 UAS-GFP*, all of which indicate ISC-like clustering either directly or indirectly. Dl staining and *ISC*^*ts*^*-Gal4 UAS-GFP* mostly coincide in contrast to Dl staining with *Dl-Gal4 UAS-GFP* that coincides less, suggesting that Dl staining and *ISC*^*ts*^*-Gal4 UAS-GFP* are better in detecting “true” ISC-like cells than *Dl-Gal4 UAS-GFP*. A benefit of using Dl staining is the nuanced protein expression it exhibits at the cell membrane as well as in intracellular puncta (vesicles). Vesicular Dl indicates Delta endocytosis and signaling activation.^73^^,^^74^ Whether Dl is more punctual in aging flies or according to midgut region and whether this indicates higher signaling in these instances would be interesting to explore.

A significant fraction of ISC-like cells co-express ISC- and EB-specific markers and exhibit variable size and ploidy. While ISC malfunctioning at a young age may seem counterintuitive, careful inspection of single-cell RNA sequencing data reveals that the expression profiles of the ISCs and EBs are more similar to each other than any other cell type in the adult fly (including EEs and ECs) to the extent that a fraction of ISCs exhibit overlapping expression profiles with a fraction of EBs. This overlap in expression profiles is evident in young as well as in old fly midgut cells.^75^ Moreover, the populations of ISCs and EBs expand over time to the expense of mature ECs. These data corroborate our analysis of the mixed ISC-EB identity cells of increased ploidy in young flies and the development of progressive dysplasia. Interestingly, old flies constitutively activate the mTOR complex 1 (mTORC1) signaling pathway in ISCs, promoting Delta expression, increased ISC size, and the EB fate.^76^ Whether this mechanism contributes to young ISC-like cell formation and clustering will be interesting to explore.

The existence of midgut cells simultaneously expressing the Dl protein and the Su(H)GBE marker is supported by previous studies. Nevertheless, it was assumed that Dl persists in EBs and leads to persistent Notch activation and faster EC differentiation^18^ or that Delta is high in EBs in genetically manipulated flies, when the expression of the Notch pathway corepressor *groucho* is lost.^77^ Instead, we show that Notch signaling between young ISCs and EBs is not binary, that is, effective versus ineffective, but rather unstable producing not only ISCs and EBs but also stem-enteroblast mixed identity cells that aggregate and accelerate their inherent malfunctions. To explain this phenomenon, we developed position-effect variegation tool, *NotchTSS-331tubGal80,* measuring the unscheduled expression of GFP in ISCs and EBs and correlated it with the number of Dl-positive cells per hotspot midgut region. This correlation was much weaker in females likely due to *Notch* expression coming from the pairing X chromosome, which may mitigate the effect of unstable expression of the monitored *Notch* locus. Thus, clustered ISC-like cells may share a mixed ISC-EB identity due to *Notch* expression instability that culminates in sustained Dl expression. Accordingly, we report that boosting *Notch* expression in EBs, decreases ISC-like cells, attesting to an insufficient but reinforceable Notch signal in prospective EBs.

Genetic and chemical manipulation of mitosis alters the rate of ISC accumulation upon aging or infection.^52^^,^^72^^,^^78^ In our study we show that ISC-like cell clusters contain nuclei of various sizes and exhibit mitosis and endoreplication. We show that DNA replication is higher in bigger clusters of 7–16 cells compared to smaller clusters of 3–6 cells, independently of midgut age, suggesting that ISC-like cells adopt either the mitotic and/or the endoreplicative fate. Nonetheless, bigger clusters exhibit increased ploidy and mitosis compared to clusters smaller than 6 cells. Interestingly, polyploid colon cancer cells exhibit a survival advantage over diploid cells,^79^^,^^80^ because increased ploidy may help tumor cells to tolerate DNA damage and chemotherapy treatments.^81^^,^^82^ Whether ISC-like cells survive better by evading apoptosis or by bypassing cell cycle checkpoints, remains to be elucidated.

To maintain genome integrity, cells go through cell cycle checkpoints and activate the DDR pathway upon DNA damage or the DRS pathway when unable to keep up with DNA synthesis.^83^ Instead of DNA damage, our data indicate spontaneous DRS signal in young flies that increases over time along with ISC-like cell clustering. We propose that γH2AvD and ATM staining mostly mark DRS in the midgut because: (1) staining is relatively uniform in the nucleus compared to DDR staining that would appear as sporadic nuclear foci; (2) big proportions of young fly midgut progenitors and enterocytes are stained; and (3) staining is intensified by DRS-inducing drugs.

DRS and ISC-like cell clustering are causally linked since genetic and pharmacological manipulation of DRS alter ISC-like cluster number and size, indicating that high DNA replication and DRS drive cluster expansion, while lower DNA replication may be important to avoid excessive stress and cell misdifferentiation.^84^^,^^85^ Mechanistically we find that *CycE* operates in a positive feedback loop between DRS and ISC-like clustering. Earlier studies involved CycE in dysplasia^52^ and its synergistic action with *Notch* inactivation in progenitors to initiate intestinal tumorigenesis.^86^ Similarly, boosting ISC division via *CycE* and *string* overexpression induces a higher frequency of loss-of-heterozygosity.^35^^,^^36^ Here we show that *CycE* induces DRS via mitosis and endoreplication, and vice versa DRS induces *CycE*. This loop promotes spontaneous ISC-like clustering in young flies and predisposes for tumor formation later in life, likely by rendering the midgut increasingly more mitogenic and prone to DNA replication errors.

We also find that the posterior P1 and P4 regions are hotspots for ISC-like cluster and tumor formation compared to the coldspot A1 region, in agreement with previous reports.^18^^,^^36^ However, alternative gut regions may be more conducive to invasive tumor formation. For instance, the synergistic effect of Apc mutation and Ras^V12^ overexpression induces clonally expanding invasive tumors specifically in the A1 region.^87^ Those clones do not express any of the typical midgut cell markers and are likely novel EB-like cells that fail to properly differentiate. Similarly, Ras^V12^ overexpression in the hindgut, but not the midgut, ECs transforms and renders them invasive.^88^^,^^89^

Prompted by the differential chromatin accessibility assigned to genes of the Notch, TGF-beta and other mitogenic and cell differentiation pathways, we propose that ISC-like cells cluster in young *Drosophila* midguts due to unstable expression of *Notch* signaling network genes in ISC progeny. We acknowledge three levels of strength in *Notch* expression and signal reception by the prospective EBs (Figure 8L): (1) efficient or strong signaling securing the EB to EC fate, (2) essentially absent signaling yielding ISC clusters, and (3) unstable or inefficient Notch signaling that traps the prospective EBs in a mixed ISC-EB fate. Therefore, rather than acting in a binary, effective-versus-non-effective Notch signal reception, prospective EBs are inherently susceptible to inadequate signaling in terms of strength or duration. This inevitably entails high ISC division rate rendering EBs produced at a fast pace prone to short-lasting Notch signaling.^90^^,^^91^ However, this mechanism of mis-differentiated cell production provides the opportunity for conceptually simple remedies by either boosting the strength of Notch signaling or by slowing down ISC division to prolong Notch signaling reception. While stem cells of different tissues and species rely on distinct signals to define their fate, the concept of suboptimal signaling in young healthy tissues producing progenitors of mixed type identity may be broadly applicable.

### Limitations of the study

We show that Notch signaling between prospective ISCs and EBs is flawed, producing ISC-like cells that cluster and compromise intestinal homeostasis and overall aging. Reinforcing Notch signaling reduces ISC-like cell clustering and alleviates old age pathologies. Moreover, Notch protein expression varies among ISCs and EBs and Notch promoter locus appears transcriptionally unstable. However, Notch signaling is regulated at multiple levels, including ligand and receptor expression and processing, negative feedback looping, an interplay between Notch and Dpp/BMP signaling, the Par complex, integrins, and Numb and Sara endosomes. To mechanistically dissect Notch signaling imperfection further studies are required. One key issue to resolve is if variability in Notch expression rather than Notch activation is the cause of compromised signaling.

## Resource availability

### Lead contact

Further information and requests for resources and reagents should be directed to and will be fulfilled by the lead contact, Yiorgos Apidianakis (apidiana@ucy.ac.cy).

### Materials availability

Drosophila strains and plasmids generated in this study are available upon request from the lead contact.

### Data and code availability

• Data: All data reported in this paper will be shared by the lead contact upon request. ATAC sequencing data have been deposited at the NCBI BioProject database and are publicly available as of the date of publication. Accession number is listed in the key resources table.
• Code: This paper does not report original code.
• All other requests: Any additional information required to reanalyze the data reported will be shared by the lead contact upon request.

## Acknowledgments

We thank Allison Bardin for advice on the construction of the NotchTSS-331tubGal80 fly line, and funding from the Republic of Cyprus “Restart 2016–2020 Programmes” through the 10.13039/501100018877Research and Innovation Foundation (LDDTA Project: EXCELLENCE/0421/0323).

## Author contributions

C.N., S.T., M.K., M.S., V.Y., K.S., and Y.A. designed the experiments. C.N., S.T., M.K., M.S., V.Y., and P.G. performed the experiments. All authors contributed to the analysis of the data. M.S., V.Y., K.S., and P.G. provided research support. C.N. and Y.A. wrote the manuscript. All authors edited the manuscript.

## Declaration of interests

The authors declare no competing interests.

## STAR★Methods

### Key resources table

**Table:** 

REAGENT or RESOURCE	SOURCE	IDENTIFIER
**Antibodies**		

Rabbit anti-phospho Histone H3(Ser10), Mitosis Marker	Millipore	06-570; RRID: AB_310177
Chicken anti-GFP	Invitrogen	A10262; RRID: AB_2534023
Rabbit anti-GFP	Invitrogen	A6455; RRID:AB_221570
Mouse anti-Prospero	DSHB	MR1A; RRID:AB_52844
Mouse anti-Delta	DSHB	C594.9B; RRID:AB_528194
Mouse anti-Notch ECD	DSHB	C458.2H; RRID:AB_528408
Rabbit polyclonal Histone H2AvD (ps137)	Rockland Immunochemicals	600-401-914
Rabbit monoclonal Phospho-ATM/ATR Substrate Motif [(pS/pT) QG]	Cell Signaling Technologies	6966; RRID:AB_10949894
Alexa Fluor™ 488 donkey anti-rabbit IgG	Invitrogen	A21206; RRID:AB_2535792
Alexa Fluor™ 488 goat anti-chicken IgG	Invitrogen	A32931; RRID:AB_2762843
Alexa Fluor™ 555 donkey anti-mouse IgG	Invitrogen	A31570; RRID:AB_2536180
Alexa Fluor™ 555 donkey anti-rabbit IgG	Invitrogen	A31572; RRID:AB_162543

**Chemicals, peptides, and recombinant proteins**		

Drosophila Agar Type II	Apex	66-103
Cornmeal	Local market	N/A
Inactivated dried yeast	Local market	N/A
Sucrose	Local market	N/A
Tegosept	Apex	20-258
Propionic acid	Scharlau	AC18941000
QIAzol® Lysis Reagent	QIAGEN	79306
Bovine Serum Albumin	Sigma	A7888
Triton™ X-100	Sigma	T8787
DAPI	Sigma	D9542-10mg
Vectashield Antifade Mounting Medium	Vector	H-1000
KAPA SYBR Fast Master Mix (2x) Universal	KAPA Biosystems	KK4602
UltraPure Distilled Water DNase/RNase Free	Invitrogen	10977-035
Ethanol absolute	Scharlau	ET00072500
Methanol	Honeywell	34860
2-Propanol	Honeywell	24137
Chloroform	Millipore	1.02445.2500
Propidium Iodide	ThermoFisher Scientific	P3566
0.5% Trypsin/EDTA	ThermoFisher Scientific	10779413
Schneider’s Insect Medium	Sigma-Aldrich	S0146
Fetal Bovine Serum	ThermoFisher Scientific	A5209401
DMSO	Sigma Aldrich	67-68-5
Hydroxyurea	Sigma Aldrich	H8627
Gemcitabine	Sigma Aldrich	G6423
Rapamycin	Alfa Aesar	J62473.X3
Floxuridine	Sigma Aldrich	F0503

**Critical commercial assays**		

RQ1 reagent kit	Promega	
PrimeScript RT Master Mix	Takara	RR036A
Click-iT™ Plus EdU Cell Proliferation Kit for Imaging, Alexa Fluor™ 555 dye	ThermoFisher Scientific	C10638

**Experimental models: Organisms/strains**		

*Drosophila melanogaster*: *w*^*1118*^	Bloomington stock center	6326; RRID:BDSC_6326
*Drosophila melanogaster*: *Oregon-R*	Bloomington stock center	2376; RRID:BDSC_2376
*Drosophila melanogaster*: DGRP-392	Bloomington stock center	28194; RRID:BDSC_28194
*Drosophila melanogaster*: DGRP-646	Bloomington stock center	28217; RRID:BDSC_28217
*Drosophila melanogaster: w; esg- Gal4 UAS-GFP tub-Gal80*^*ts*^	Michelli and Perrimon^10^	NA
*Drosophila melanogaster: esg-Gal4 UAS-GFP; Su(H)-Gal80 tub-Gal80*^*ts*^	Zeng and Hou^8^	NA
*Drosophila melanogaster: w; Su(H)-Gal4 UAS-CD8GFP tub-Gal80*^*ts20*^*/CyO* (Su(H)^ts^)	Zeng et al.^55^	NA
*Drosophila melanogaster: w; UAS-srcGFP/CyO; Dl-Gal4/TM6C*	Zeng et al.^55^	NA
*Drosophila melanogaster: yw; If/CyO; Su(H)GBE-nlsGFP/TM6B*	De Navascués et al.^92^	NA
*Drosophila melanogaster: ry506 Dl-lacZ05151/TM3*	De Celis et al.^54^	NA
*Drosophila melanogaster: Myo1A-Gal4 UAS-GFP;Su(H)GBE-Gal80 tubGal80*^*ts20*^	This paper	NA
*Drosophila melanogaster: Actin5C-Gal4 UAS-srcGFP/CyO*	Evangelou et al.^93^	NA
*Drosophila melanogaster: NotchTSS-331tubGal80*	This paper	NA
*Drosophila melanogaster:* *UAS-His2Av*	Vienna Drosophila Resource Center	v110598
*Drosophila melanogaster:* *UAS-spn-B*	Vienna Drosophila Resource Center	v105623
Drosophila melanogaster: *w; UAS-CycE*	Bloomington Drosophila Stock Center	4781; RRID:BDSC_4781
*Drosophila melanogaster: w; UAS-CycE RNAi*	Vienna Drosophila Resource Center	v110204
*Drosophila melanogaster: UAS-NotchDN*	Apidianakis et al.^51^	NA
*Drosophila melanogaster: UAS-Notch*^*RNAi*^	Bloomington stock center	7078; RRID:BDSC_7078
*Drosophila melanogaster: UAS-Notch*^*IC5*^	Go et al.^94^	NA
*Drosophila melanogaster: UAS-InR*	Bloomington stock center	8262; RRID:BDSC_8262
*Drosophila melanogaster: UAS-hep*^*wt*^	Bloomington stock center	9308; RRID:BDSC_9308
*Drosophila melanogaster: PBac{y[+mDint2] GFP[E.3xP3]=vas-Cas9}VK00027*	Bloomington stock center	51324; RRID:BDSC_51324
*Drosophila melanogaster: y[1] w[67c23] P{y[+mDint2]=Crey}1b; sna[Sco]/CyO; Dr[1]/TM3*	Bloomington stock center	34516; RRID:BDSC_34516

**Oligonucleotides**		

See Table S2 for primer sequences	Integrated DNA Technologies (IDT)	NA

**Recombinant DNA**		

pHRTC_Lw_tubGal80	pUC19 plasmid with SV40<*Gal80*<tubulin-promoter, loxP>hsp70-promoter>*white*>loxP	This paper
pCFD3-dU6:3sgRNA plasmid	Port et al.^95^	RRID:Addgene_49410

**Software and algorithms**		

ImageJ	NIH	https://imagej.nih.gov/ij/
Prism 9	GraphPad	https://www.graphpad.com/scientific-software/prism/
Microsoft Office 2021	Microsoft	https://www.office.com/
Mann-Whitney U Test Calculator	Social Science Statistics	www.socscistatistics.com

**Deposited data**		

ATAC-Seq data	https://www.ncbi.nlm.nih.gov/bioproject/	PRJNA1217363

### Experimental model and study participant details

#### *Drosophila* stocks

Gal4 lines: ISCs and EBs expression, *w; esg- Gal4 UAS-GFP tub-Gal80*^*ts*^ (esg^ts^)^10^; for ISC expression, *esg-Gal4 UAS-GFP; Su(H)-Gal80 tub-Gal80*^*ts*^ (ISC^ts^)^8^ and *w; UAS-srcGFP/CyO; Delta-Gal4/TM6C*^55^; for EB expression, *w; Su(H)-Gal4 UAS-CD8GFP tub-Gal80*^*ts20*^*/CyO* (Su(H)^ts^)^55^ and for EC expression, *tub-Gal80*^*ts*^*/FM7; Myo1A-Gal4 UAS-EGFP/CyO* (MyO^ts^).^51^ The reporters *yw; If/CyO; Su(H)GBE-nlsGFP/TM6B*^92^ and *ry506 Dl-lacZ*^*05151*^*/TM3* were used to assess ISC-like cells identity. UAS-His2Av^RNAi^ #v110598, UAS-spn-B^RNAi^ #v105623 and UAS-CycE^RNAi^ #110204 were obtained from the Vienna Drosophila Resource Center (VDRC) and UAS-Notch^RNAi^ #7078 from Bloomington Stock Center (BDSC). Additional stocks obtained from BDRC: UAS-InR #8262, UAS-hep^wt^ #9308, UAS-CycE #4781 and the two inbread strains of the Drosophila Genetic Reference Panel (DGRP) collection; DGRP-392 #28194 and DGRP-646 #28217. Other stocks used in this study include the UAS-Notch^DN 51^ and UAS-Notch^IC5 94^. Tubulin-G80 (generated for this study-see below) was backcrossed to *w; Actin5C-Gal4 UAS-srcGFP/CyO* (originating from BDSC #25374 and BDSC #5432 ^93^. *w*^*1118*^ was used as a control for all UAS-transgenic flies and Oregon-R as a typical wild-type strain. We used only adult *Drosophila* of specified sex, genotype and age as mentioned in the corresponding Figure legends.

#### *Drosophila* rearing

All stocks were routinely maintained at 18°C or 25°C on a 12:12h light:dark cycle on a standard flyfood medium: 10 g agar, 60 g cornmeal, 30 g yeast, 50 g sugar, 5.8 ml of a 20% Tegasept dissolved in 100% ethanol and 3.8 ml 99% propionic acid for 1 Lt of flyfood. Yeast-restriction media: standard medium was modified to contain 10%, 2% or 0.5% of yeast. For yeast-restriction experiments flies were fed on yeast-restriction media for 4-, 30- and 40-days at 25°C.

For ISC-like cell cluster assessment during aging, ISC^ts^, *Dl-lacZ*^*05151*^ and *Dl-Gal4 UAS-GFP* lines were outcrossed to Oregon-R at 25°C and adults remained for 4, 30 or 42 days at 25°C. For ISC-like cell identity experiments flies were reared and remained at 25°C for 30 days. ISC^ts^ outcrossed to *w*^*1118*^ were reared at 18°C and adults were transferred at 29°C for up to 21days.

GAL4-UAS^96^ crosses were reared at 18°C and female adult flies (3-5 days) were transferred at 29°C to induce the transgenes before experiments. His2Av^RNAi^ and spn-B^RNAi^ were induced for up to 21days at 29°C. CycE^RNAi^ and CycE^OE^ were induced for 7 days, Notch^DN^ and UAS-Notch^RNAi^ for 3 days at 29°C, Notch^IC5^ for 4 and 30 days at 25°C, InR and UAS-hep^wt^ at 29°C for 1, 3 and 6 days. Details for generation of tumorous flies are given below. *NotchTSS-331tubGal80* flies were crossed to esgts and ISC^ts^ at 18°C and transferred at 29°C for 7 and 14 days.

For spontaneous tumorigenesis outcrossed and backcrossed *Dl-Gal4 UAS-GFP* flies were incubated at 25°C for 42 days. For tumorigenesis induced by Notch downregulation *esg*^*ts*^*-Gal4 UAS-GFP* flies were crossed to UAS-Notch^DN^. Progeny were collected and let to mature at 18°C for 5-7 days, and transferred to 29°C for 2-, 4- and 8-days (females) or 4-, 8- and 12-days (males).

For MARCM analysis *w hs-FLP tub-Gal4 UAS-nlsGFP/FM7; FRT82B tub-Gal80/TM6B*^97^ were crossed to *w; FRT82B arm-lacZ/TM6B* (BDSC# 7369) at 25°C. Progeny were collected, heat shocked for 60 minutes at 37°C for clone induction, and let to recover for 1, 3, 7 14 and 20 days at 25°C. 10 midguts were immunostained and number of clones and cells per clone per midgut were quantified using a Zeiss Axioscope A.1 fluorescence microscope.

### Method details

#### Construction of the *NotchTSS-331tubGal80* fly line via CRISPR/Cas9

To PCR amplify the remote (left, LHA) and proximal (right, RHA) homology arm upstream of *Notch* TSS the following primer pairs were used (genomic site#1 in capitals; cloning adaptors in lower case):

LHA-Foreword, 5′tacccggggatccACGAAACCGAAAATCAATTCAATTATATAC3′ and

LHA-Reverse, 5′tatcatgtctggatACTTGTAGCATTTTTTAAGTATTTTATTTTCC3′.

RHA-Foreword, 5′aagatctccatgGCGTTTTTCAATCAAATTTATGC3′ and

RHA-Reverse, 5′ttacgccaagcttGATCATCTTATCTCATAGTTTTGG3′.

To generate the donor plasmid (pHRTC_Lw_tubGal80_Notch_site#1), LHA, RHA and a tubGal80 transgene cassette inserted into a pUC19 plasmid were recombined via Gibson assembly in the following order: (i) LHA, (ii) SV40<*Gal80*<*tubulin*-promoter, loxP>*hsp70*-promoter>*white*>loxP, and (iii) RHA.

The resulted transgene was guided upstream of *Notch* via a “pCFD3-dU6:3sgRNA” plasmid (Addgene, #49410) containing a sgRNA complementary to a seed sequence 149bp upstream of *Notch* TSS (seed sequence, PAM sequence underlined, 5′-AACTACTCATGCAAGCGGCTCGG-3′).

Fly transgenics were produced at Bestgene Inc (CA, USA) using a mixture of sgRNA and donor plasmid to inject fly embryos expressing Cas9 (BDSC#51324 PBac{y[+mDint2] GFP[E.3xP3]=vas-Cas9}VK00027). The *white* gene marker in the donor vector was used to select and balance transgenics in parental and F1 generations. The loxP-flanked sequence surrounding the *white* gene was subsequently removed by crossing to flies that expressed Cre recombinase (BDSC#34516 *y[1] w[67c23] P{y[+mDint2]=Crey}1b; sna[Sco]/CyO; Dr[1]/TM3*).

We confirmed the resulting transgenics by genotyping PCR using multiple primer pairs in and out of the insertion. For example, PCRtest1 (forward 5′-GAGCACTAAGAATGTGACTGCTTTCGTTTGT-3′ and reverse 5′-ATGGGAGCAGTGGTGGAATGCC-3′) and PCRtest2 (forward 5′TTGCAGAGGCCAGGGCAATG-3′ and reverse 5-’TGCGGCACAACACAGCGT-3′). Sequencing results verified insertion 331bp upstream of the *Notch* TSS.

#### Drug administration

Hydroxyurea (HU, Sigma#H8627) and Gemcitabine (Sigma# G6423) were dissolved in ddH_2_O and added in flyfood at a concentration of 1 mg/ml and 30μM, respectively. Rapamycin (Alfa Aesar #J62473.X3) and floxuridine (Sigma#F0503) were dissolved in 100% ethanol and ddH_2_O, respectively, and added in flyfood at a concentration of 200μM.

Flyfood was reheated in a microwave and 2 mL was aliquoted in fly vials and thoroughly mixed with 1.8μl of 33.4mM Gemcitabine in water, 40μl of 50 mg/ml HU in water, 20μl of 18.28 mg/ml Rapamycin in ethanol or 20μl of 4.92 mg/ml floxuridine in water. Mock treatments were prepared by adding the corresponding amount of solvent.

Feeding HU and Gemcitabine: 5–7-day old females raised at 18°C were transferred in groups of 10-15 in standard flyfood at 29°C for 2 days to allow GFP expression and then for 2 more days in drug containing flyfood.

Feeding Rapamycin: 3–5-day old females raised at 2°C were transferred in groups of 10-15 in drug containing flyfood at 25°C for the first 4 days (4-day old flies), or between days 21-30 or 30-40 days (30- and 40-days old, respectively). To assess DRS upon Rapamycin feeding, flies were continuously fed on the drug for up to 21 days.

Feeding Floxuridine: same as for Rapamycin except that long term feeding lasted for the first 5 days or the last 10 days of their life. To assess DRS upon Floxuridine feeding, flies were fed on the drug for the first 5 days and then transferred on standard food.

#### Progenitor cell isolation (FACS)

Cell dissociation and FACS protocol is that described by^98^ with minor modifications. To isolate ∼50,000 GFP-positive alive cells for each replicate sample, the anterior (A1- A3) and posterior (P1-P4) midgut regions were manually dissected from 120 female and 210 male 4-day old esg^ts^ flies in 1X PBS and dissociated using 0.5% Trypsin/EDTA (ThermoFisher) for 30 minutes at room temperature (RT) with mild shaking. Dissociated cells were collected at regular 30 minutes intervals, resuspended in 1X PBS/1% Bovine Serum Albumin (BSA) and kept on ice. Prior to FACS sorting, dissociated cells were resuspended in fresh 1X PBS/1% BSA solution containing 1 mg/mL Propidium Iodide (PI) marker for live/dead cell separation. Alive progenitor cells (GFP^+^ PI^-^) were sorted using the S3e Cell Sorter (Bio-Rad). Sorted cells were frozen in 70% Schneider’s Insect Medium (S0146, Sigma-Aldrich), supplemented with 20%FBS and 10%DMSO and stored at -80°C degrees until shipped for ATAC-Seq analysis to Active Motif.

#### ATAC-seq assay

Cryopreserved cells were thawed in a 37°C water bath, pelleted, washed with cold PBS and tagmented as previously described in,^99^ with some modifications based on.^100^ In short, cell pellets were resuspended in lysis buffer, pelleted, and tagmented using the enzyme and buffer provided in the ATAC-Seq Kit (Active Motif). Tagmented DNA was then purified using the MinElute PCR purification kit (Qiagen), amplified with 10 cycles of PCR, and purified using Agencourt AMPure SPRI beads (Beckman Coulter). Resulting material was quantified using the KAPA Library Quantification Kit for Illumina platforms (KAPA Biosystems), and sequenced with PE42 sequencing on the NovaSeq 6000 sequencer (Illumina).

#### Dissections and immunohistochemistry

Dissections of adult midguts and immunohistochemistry were performed as previously described.^51^ Flies from each genotype, were dissected on Sylgard (VWR) plates in 1× PBS (130mM NaCI, 70mM NA_2_HPO4, 30mM NaH_2_PO_4_) and fixed in 4% formaldehyde (FA, Polysciences) in 1x PBS for 20 minutes at RT. Three quick rinses were performed with 1× PBS. Tissues were incubated in blocking PBT solution (1× PBS, 0.2% Triton-Χ, 0.5% BSA) at RT for at least 20 minutes. Tissues were incubated with primary antibodies diluted in PBT overnight at 4°C, washed 3 times in RT for 10 minutes with PT (1x PBS, 0.2% Triton X-100), incubated in secondary antibodies diluted in PBT (with DAPI when is required) for 1-2 hours at RT with mild shaking, washed 3 times as before and mounted in Vectashield (Vector Laboratories). Primary antibodies: rabbit anti-pH3 (Millipore 1:4000;), mouse anti-Prospero (DSHB 1:100), chicken anti-GFP (Invitrogen 1:2000) and rabbit anti-GFP (Invitrogen 1:3000). Secondary antibodies: donkey anti-rabbit, donkey anti-mouse conjugated to Alexa 555(Invitrogen, 1:1000); donkey anti-rabbit, goat anti-chicken conjugated to Alexa 488 (Invitrogen, 1:1000), DAPI used to stain DNA (Sigma 1:4000 of 10 mg/ml stock). For staining with mouse anti-Delta (Dl, DSHB 1:100), anti-Notch (NECD, DSHB 1:100), anti-Pdm1 (Nub, DSHB 1:100), and rabbit anti-H2avD (Rockland Immunochemicals 1:1000) and anti-phospho-ATM/ATR (ps/pT) (Cell Signaling Technologies 1:500) an additional fixation step with methanol was performed.^101^ Briefly, following the last wash with 1xPBS of the primary fixative, cold methanol was added dropwise in 2:1 dilution (methanol:1xPBS), then replaced with 100% methanol and incubated for 5 minutes at RT, then 1xPBS was added dropwise in 2:1 dilution (1xPBS:methanol), then removed and rinsed twice with 1xPBS.

#### EdU assay

For EdU staining, a protocol for the Click-iT EdU Alexa Fluor 555 Imaging Kit (ThermoFisher Scientific, C10638) was used. 4-days and 14-days old female flies (ISC^ts^>GFP) were transferred to a vial containing 1 mg/mL EdU in 20% sucrose for 24 hr at 29°C degrees. Whole guts were dissected and fixed in 4% FA for 20 minutes at RT. The rest of the procedure was performed according to manufacturer’s instructions.

#### RNA isolation and RT-qPCR

Quantification of mRNA expression levels, was performed by dissecting 25 midguts per genotype per biological replicate. Total RNA was extracted using Qiazol regent (Qiagen) and dissolved in RNase-free water. 800 ng of total RNA were freed from genomic DNA by the RQ1 RNase-Free DNase Kit (Promega). Reverse transcription was performed using 145,4ng of the total DNAse-treated RNA using the TaKaRa PrimeScript RT Master Mix Kit. qPCR reactions were performed using gene specific primers (Table S1) with the amplification program shown in Table S2, using the Bio-Rad CFX Manager 3.1 program. Gene expression was normalized to the expression of two references genes, *rpL32* and *α-tub* using the 2-ΔΔCt method. ≥3 biological replicates were used to calculate the mean and the standard deviation.

### Quantification and statistical analysis

#### Image acquisition and analysis

MARCM clones, pH3-positive, *ISC*^*ts*^*>GFP*-positive and Dl-positive cells and ISC tumors were measured under the fluorescent microscope (Zeiss Axioscope A.1 and Nikon Eclipse Ti) at 20 × magnification along the whole midgut or region specifically. ISC-like cells were defined as clusters of 3-20 ISC marker positive cells that are heterogenous in nucleus size. ISC tumors were defined as clusters of >80 ISC marker positive cells homogenous in nucleus size.

All the images shown, are stacks of optical sections acquired using the Leica TCS SP2 DMIRE2 confocal microscope captured at 63x magnification, zoom 1× (unless otherwise noted) and 1024×1024 format and produced as a maximum projection of 10-12 serial optical sections. Images compared were acquired using the same settings. Every experiment measuring γH2Av or ATM/ATR (pS/TQ) was performed under the acquisition parameters based on the strongest signal obtained to avoid using images exhibiting signal saturation.

For γH2Av and pS/TQ signal quantification within ISC-like cell clusters, ≥3 images from each frame of 237.5x 237.5μm per region were analyzed in Image J. Specifically, the different channels were separated and each cell population was selected and measured independently. Signal values (mean gray value) minus average background fluorescence were multiplied by the number of pixels per cell to generate integrated density values. The latter were plotted using Prism 9 and the Student’s t-test was used for statistical analysis.

For γH2Av and pS/TQ signal quantification per midgut area, ≥3 images per condition were used manually highlighting the surface of each midgut in each frame using the Freehand selection tool of the software. γH2Av intensity in proximity to tumor was measured using 20 individual cells either located inside the tumor, attached to tumor, 3-6 EC away from tumor, or >6 ECs away from the tumor.

DNA content measurements were performed as previously described.^52^ Briefly, 3 images were acquired at the same settings, scanning throughout the DAPI stained nuclei. Sum projections of 10-12 serial optical sections were used to measure the total DAPI fluorescence in the P4 region of 7 days old females, using Image J. Specifically, all *ISC*^*ts*^*>GFP*-positive cell nuclei (from single ISCs and clustered ISC-like cells) were selected manually using the oval selection tool and subsequent measurements for nucleus maximum cross-section area and integrated density were acquired. The DAPI signal of each nucleus was normalized to the mean signal of 12 small, single, *ISC*^*ts*^*>GFP*-positive cells. The normalized data points of many cells arising from the same region (P4) of 3 midguts were added together.

Increase in Dl-positive cell surface area was determined according to the maximum size of a population of 20 single Dl-positive cells selected from 3 different images of the A1 region of 4 days old flies using ImageJ. The larger surface area among these measurements (23μm^2^) was used as a threshold.

To calculate the % of *ISC*^*ts*^*>GFP*-positive cells that were also Dl-positive and vise-versa, ≥3 images from each frame of 237.5x 237.5μm per midgut region, per time point were used.

Correlations of GFP-positive with Dl-positive and Pros-positive cells of *NotchTSS-331tubGal80 Gal4/UAS-GFP* flies were analyzed in Image J using ≥10 images of 237.5x 237.5μm per midgut region.

For EdU cell counting analysis, confocal images of posterior (P1, P4) midgut were taken from all samples. EdU-positive cells were quantified only in GFP-positive clusters and the % of EdU-positive cells in clusters was calculated.

#### ATAC-seq analysis

Reads were aligned using the BWA algorithm (mem mode; default settings). Duplicate reads were removed. Only reads mapping as matched pairs and only uniquely mapped reads (mapping quality ≥1) were used for further analysis. Alignments were extended in silico at their 3′-ends to a length of 200 bp and assigned to 32-nt bins along the genome. The resulting histograms (genomic “signal maps”) were stored in bigWig files. Peaks were identified using the MACS 2.1.0 algorithm at a cutoff of p-value 1e-7, without control file, and with the nomodel option. Signal maps and peak locations were used as input data to Active Motif’s proprietary analysis program, which creates Excel tables containing detailed information on sample comparison, peak metrics, peak locations, and gene annotations. Images of peak annotations were taken and edited from UCSC Genome browser (https://genome.ucsc.edu/index.html). KEGG pathway analysis was performed using GeneCodis online tool (https://genecodis.genyo.es/) and curated to remove falsely attributed genes.

#### Statistical analysis

Prism9 was used for: (i) two-tailed Student’s t-test to compare pairwise groups of at least 6 values per group, (ii) for one way ANOVA for multiple comparisons and/or n=3-6, and (iii) Pearson correlation and linear regression. Significance in correlation was determined using linear regression (R^2^) and n≥10 data points. Relative mRNA levels and groups of values with n=4-7 biological replicates were assessed using the Mann-Whitney U Test Calculator (www.socscistatistics.com). Chi-square test was used to compare the number of clusters with 3 or more cells between conditions by sampling the same number of midguts (*n*=10 midguts) and expecting the same number of clusters per midgut between conditions compared. For one degree of freedom, chi-square values of >3.841, >6.635 and >10.828 correspond to *p*<0.05, *p* <0.01 and *p* <0.001, respectively. Error bars in all graphs represent standard deviation. Significance is indicated by ∗p<0.05, ∗∗p<0.01, ∗∗∗p<0.001, and ∗∗∗∗p<0.0001.; ns, not statistically significant.
