# Supplementary material for: Aberrant enterocyte progenitor clustering as an early life biomarker of Drosophila aging
Source: iScience. 2025 Feb 6;28(3):111967. doi: 10.1016/j.isci.2025.111967 (PMC11889619; doi:10.1016/j.isci.2025.111967)
Supplement: Document S1. Figures S1–S12 and Tables S1 and S2 [file mmc1.pdf]

iScience, Volume *28*

Supplemental information

Aberrant enterocyte progenitor clustering

as an early life biomarker of *Drosophila* aging

Constantina Neophytou, Savvas Teloni, Maria Koumouri, Marine Stefanutti, Panagiota Gianni, Vural Yilmaz, Katerina Strati, and Yiorgos Apidianakis

Figure S1

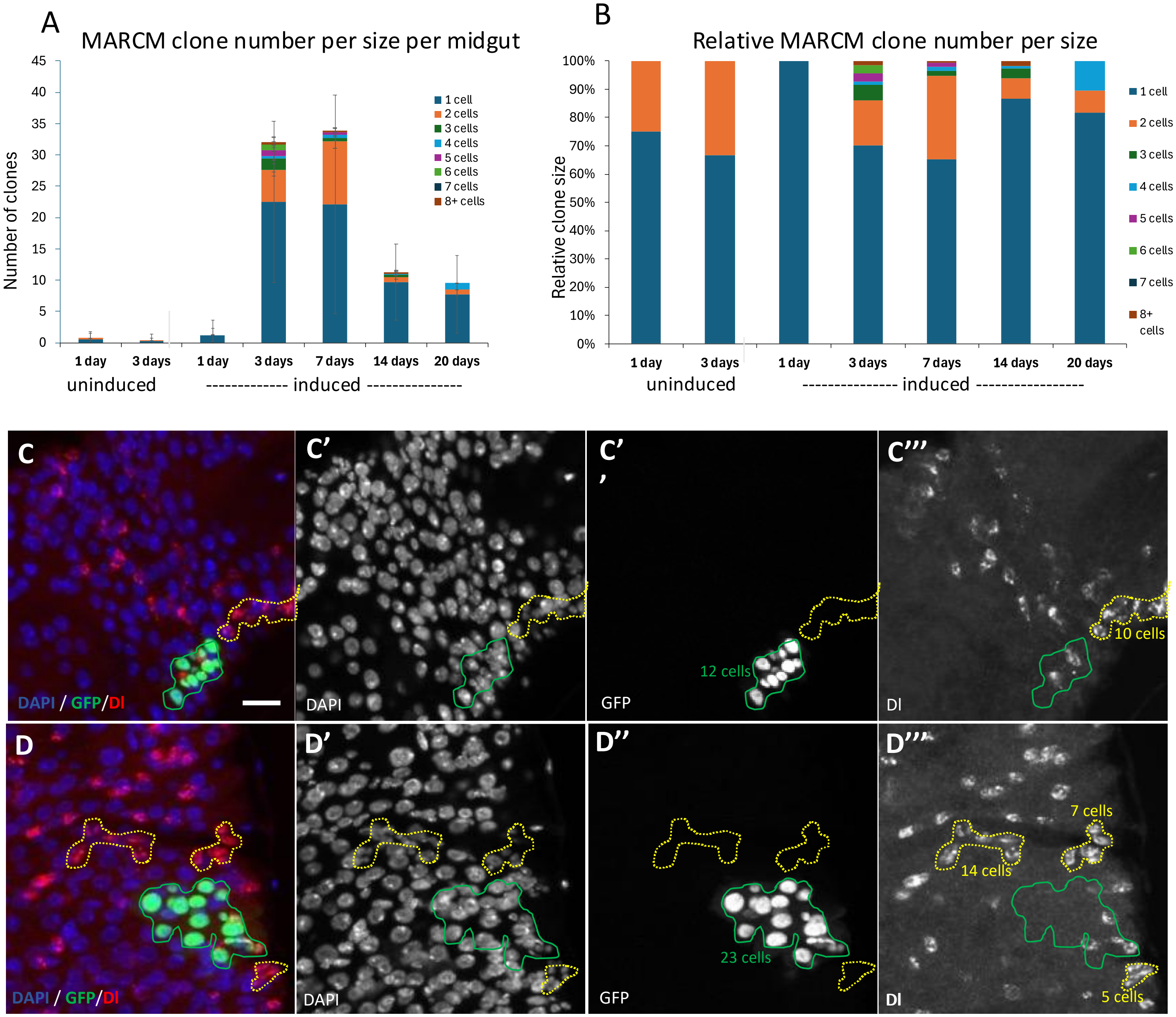

Figure S1: MARCM82B clones contain few Dl-positive cell although surrounded by sizable Dl-positive cell clusters, Related to Figure 1.

(A) Quantification of the number of MARCM82B clones per size per midgut 1, 3, 7, 14 and 20 days post induction by heat shock and controls of 1 and 3 post mock induction (uninduced).

(B) Percentage of MARCM82B clones per clone size at 1, 3, 7, 14 and 20 days post induction and induction (induced) and controls of 1 and 3 post mock induction (uninduced).

(C-D) Posterior midguts 14 days post induction containing: (C) a 12 GFP+ cell clone (within green line) and a nearby 10 cell Dl+ cell cluster (within dotted yellow line); and (D) a 23 GFP+ cell clone (within green line) and 3 nearby Dl+ cell clusters of 14, 7 and 5 cells (within dotted yellow lines). GFP shows clonally produced cells, Dl marks for ISC-like cells and DAPI stains all nuclei. Magnification is 40x zoom 2. Scale bar: 50 µm for (C-D).

Figure S2

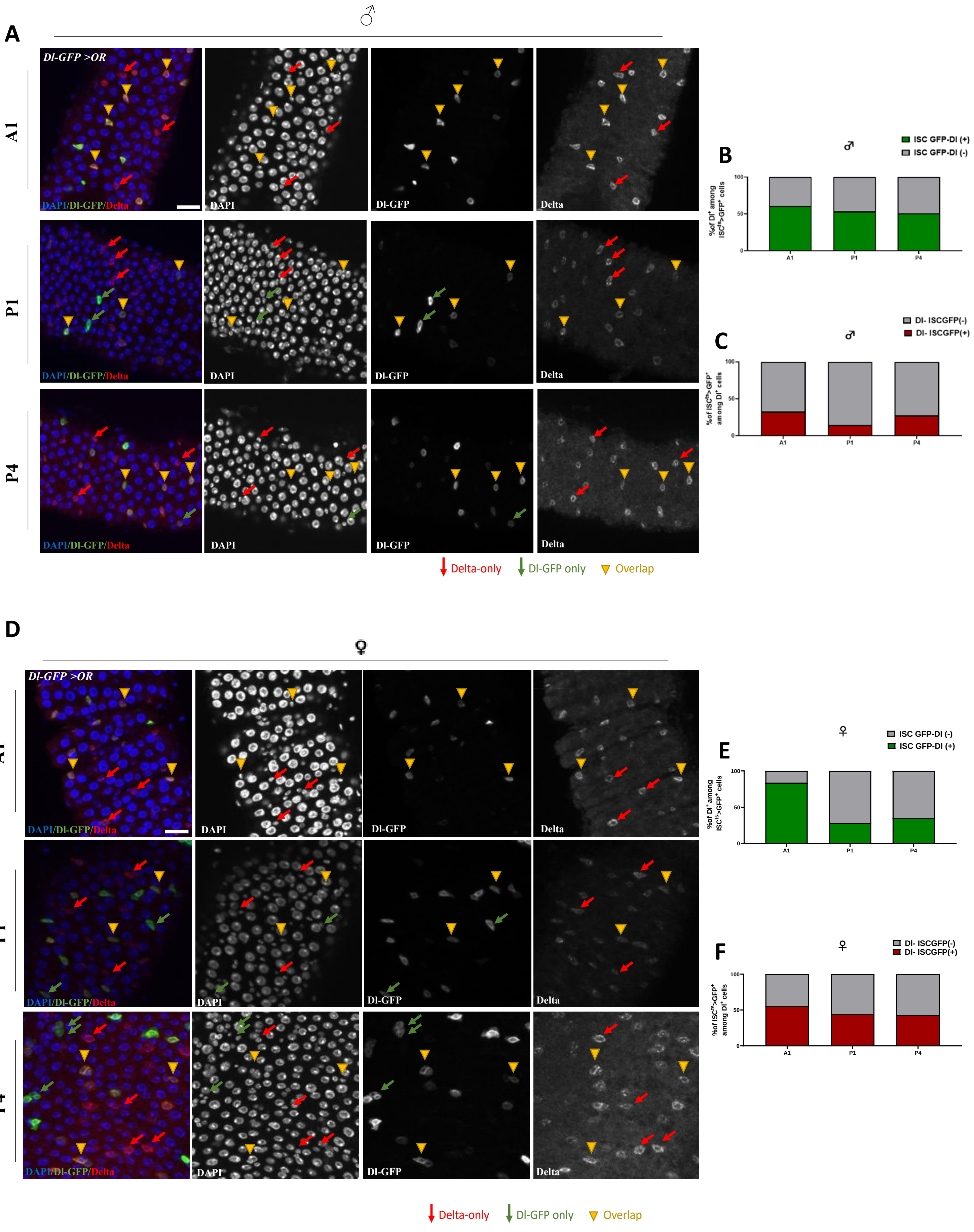
A
♂
Dl-GFP >OR
A1
P1
P4
DAPI/Dl-GFP/Delta
DAPI
Dl-GFP
Delta
B
ISC GFP-Dl (+)
ISC GFP-Dl (-)
C
Dl- ISCGFP(-)
Dl- ISCGFP(+)
100
50
0
A1
P1
P4
Delta-only
Dl-GFP only
Overlap
D
♀
E
ISC GFP-Dl (-)
ISC GFP-Dl (+)
F
Dl- ISCGFP(-)
Dl- ISCGFP(+)

Figure S2: Overlap between Dl>GFP and Dl-expressing cells, Related to Figure 2.

(A-B) *Dl-GFP* (green) and Dl-stained (red) male (A) and female (B) midgut regions, A1, P1 and P4, of a *Dl-Gal4 UAS-GFP* fly strain outcrossed to Oregon R and incubated at 25°C for 4 days. Red arrows indicate cells marked only with Dl, green arrows those marked only with GFP and yellow arrows cells marked by Dl and GFP.

(B,E) % of *Dl-GFP*-positive cells in males (B) and females (E) that are also Dl-positive (green) or Dl-negative (gray) in A1, P1 and P4 (n=4 replicates).

(C,F) % of Dl-positive cells in males (C) and females (F) that are also *Dl-GFP*-positive (green) or *Dl-GFP*-negative (gray) in A1, P1 and P4 (n=4 replicates).

Scale bar: 50 µm for (A,D).

Figure S3

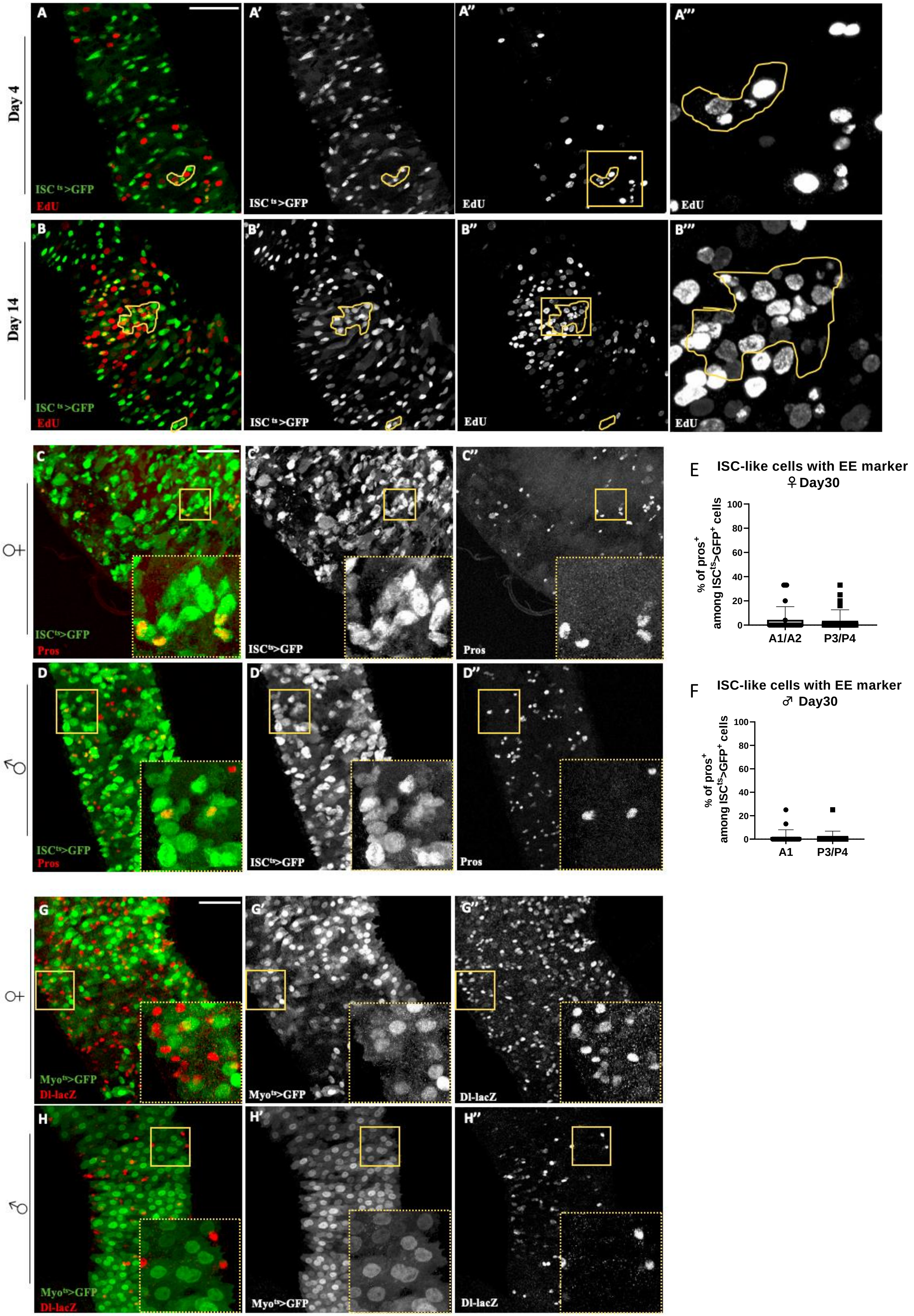
A
A'
A''
A'''
Day 4
ISC ts>GFP
EdU
ISC ts>GFP
EdU
EdU
B
B'
B''
B'''
Day 14
ISC ts>GFP
EdU
ISC ts>GFP
EdU
EdU
C
C'
C''
♀
ISCts>GFP
Pros
ISCts>GFP
Pros
D
D'
D''
♂
ISCts>GFP
Pros
ISCts>GFP
Pros
E
ISC-like cells with EE marker
♀ Day30
% of pros+ among ISCts>GFP+ cells
100
80
60
40
20
0
A1/A2
P3/P4
F
ISC-like cells with EE marker
♂ Day30
% of pros+ among ISCts>GFP+ cells
100
80
60
40
20
0
A1
P3/P4
G
G'
G''
♀
Myots>GFP
Dl-lacZ
Myots>GFP
Dl-lacZ
H
H'
H''
♂
Myots>GFP
Dl-lacZ
Myots>GFP
Dl-lacZ

Figure S3: ISC-like clusters contain EdU-positive cells of various sizes but low ISC/EE and no ISC/EC mixed identity, Related to Figure 2.

(A-B) EdU-positive cells (red in A,B; white in A'',A''',B'',B''') within *ISC^{ts}>GFP*-positive cell clusters (green in A,B; white in A',B') in the P1 region of 4- (A) and 14-day old (B) female midguts. (A''',B''') Higher magnification of EdU-positive cells of various sizes in small ISC-like clusters of 4 day old females (A''') and in bigger clusters of 14-day old females (B''').

(C-F) *ISC^{ts}>GFP*-positive cells in the P3/P4 region of females and males (green in C and D, white in C' and D') and Pros-positive pre-EEs and EEs (red in C and D, white in C'' and D''). The % of GFP=positive cells simultaneously expressing Pros (yellow in C and D) is indicative of mixed ISC/EE identity but is minimal in the A1/A2 and P3/P4 of females (E) and males (F). n=28 for (E) and n=20 for (F). One-way ANOVA for multiple comparisons was used to test significance.

(G-H) *Dl-lacZ MyO1A-Gal4 UAS-GFP/Su(H)GBE-Gal80 tub-Gal80ts* female (G) and male (H) midguts expressing GFP in ECs (green in G and H, white in G' and H') and stained for Dl-lacZ (red in G and H, white in G'' and H''). No overlap is observed between the two markers.

Scale bars: 50 µm for (A-D) and (G-H).

Figure S4

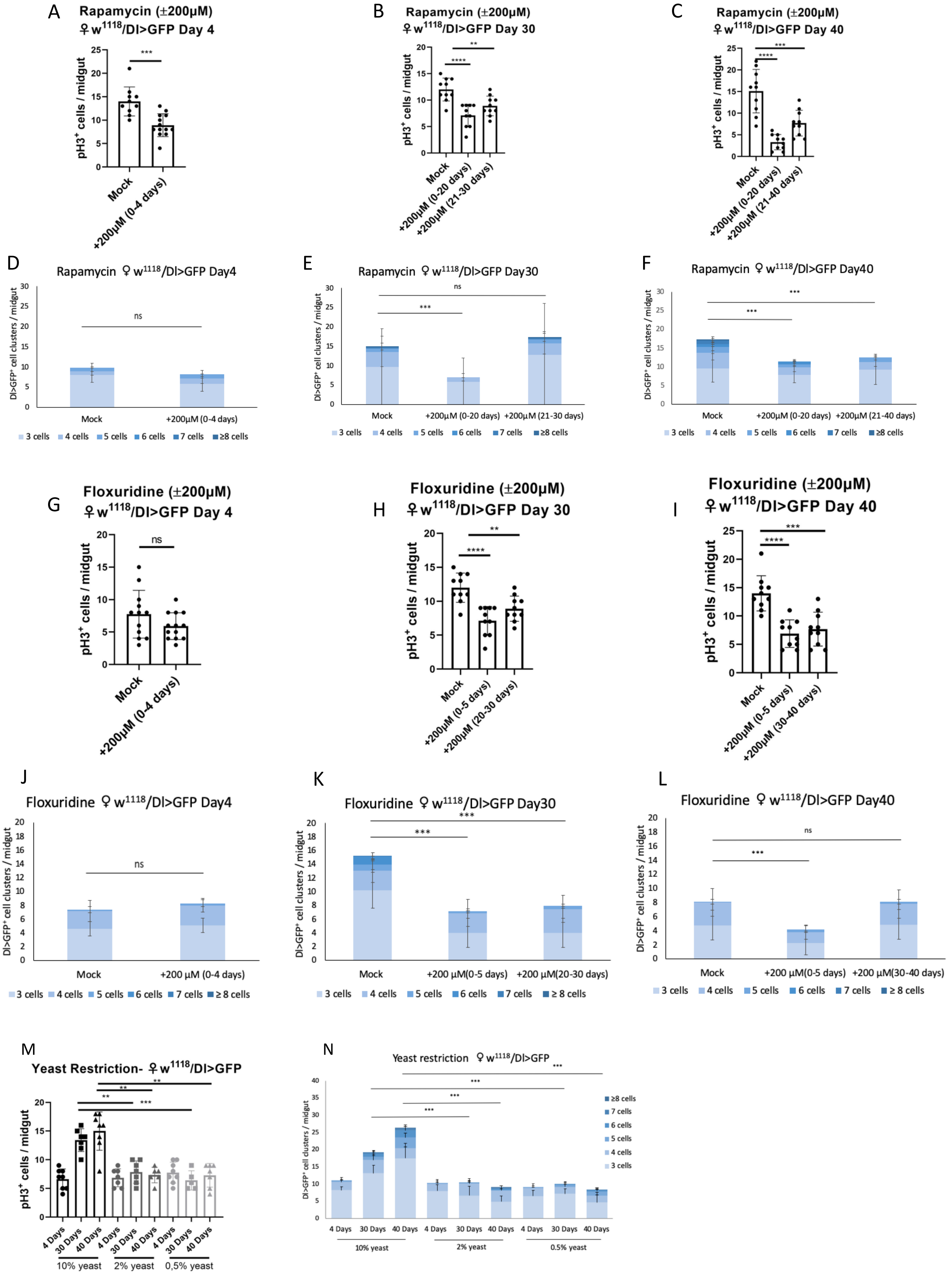
A
Rapamycin (±200μM)
♀w1118/Dl>GFP Day 4
pH3+ cells / midgut
Mock
+200μM (0-4 days)
B
Rapamycin (±200μM)
♀w1118/Dl>GFP Day 30
+200μM (0-20 days)
+200μM (21-30 days)
C
Rapamycin (±200μM)
♀w1118/Dl>GFP Day 40
+200μM (0-20 days)
+200μM (21-40 days)
D
Rapamycin ♀w1118/Dl>GFP Day4
Dl>GFP+ cell clusters / midgut
ns
+200μM (0-4 days)
3 cells 4 cells 5 cells 6 cells 7 cells ≥8 cells
E
Rapamycin ♀w1118/Dl>GFP Day30
F
Rapamycin ♀w1118/Dl>GFP Day40
G
Floxuridine (±200μM)
♀w1118/Dl>GFP Day 4
H
Floxuridine (±200μM)
♀w1118/Dl>GFP Day 30
+200μM (0-5 days)
+200μM (20-30 days)
I
Floxuridine (±200μM)
♀w1118/Dl>GFP Day 40
+200μM (0-5 days)
+200μM (30-40 days)
J
Floxuridine ♀w1118/Dl>GFP Day4
+200 μM (0-4 days)
K
Floxuridine ♀w1118/Dl>GFP Day30
+200 μM(0-5 days)
+200 μM(20-30 days)
L
Floxuridine ♀w1118/Dl>GFP Day40
+200 μM(0-5 days)
+200 μM(30-40 days)
M
Yeast Restriction- ♀w1118/Dl>GFP
4 Days 30 Days 40 Days
10% yeast
2% yeast
0,5% yeast
N
Yeast restriction ♀w1118/Dl>GFP
10% yeast
2% yeast
0.5% yeast

Figure S4: Rapamycin and floxuridine treatment and yeast restriction reduces mitosis and ISC-like clustering, Related to Figure 3.

(A-C) pH3-positive cells per midgut upon administration of 200 µM rapamycin in the flyfood of *Dl-Gal4 UAS-GFP* flies outcrossed to w^{1118}, under the parameters described in P-R. Statistical test, Student's t test using 10 midguts.

(D-F) *Dl>GFP*-positive cell clusters per midgut of flies and conditions described in A-C. Statistical test, Chi-square test using 10 midguts.

(G-I) pH3-positive cells per midgut upon administration of 200 µM floxuridine in the flyfood of *Dl-Gal4 UAS-GFP* flies outcrossed to w^{1118}. 4- (G), 30- (H) and 40-days old (I) females were treated with 200 µM floxuridine in food, either early in life (0-5 days) or later in life (Day 20-30 for H and Day 30-40 for I) and compared to mock-treated flies. Statistical test, Student's t test using 10 midguts.

(J-L) *Dl>GFP*-positive cell clusters per midgut of flies and conditions described in G-I. Statistical test, Chi-square test using 10 midguts.

(M) pH3-positive cells per midgut of *Dl-Gal4 UAS-GFP* flies outcrossed to w^{1118} feeding on 10%, 2% and 0.5% yeast for 4-, 30- and 40-days. One-way ANOVA for multiple comparisons was used to test significance, n≥6 midguts.

(N) *Dl>GFP*-positive cell clusters per midgut of flies and conditions described in M. Experiments were repeated at least twice. Statistical significance via chi-square test in (D-F), (J-L) and (N) using 10 midguts and Student's t test in (A-C), (G-I) and(M) using ≥6 midguts. Significance is indicated by *p<0.05, **p<0.01, ***p<0.001, and ****p<0.0001; ns, not statistically significant.

Figure S5

A

Signaling Pathways	♀ Anteroposterior Differential Accessbility	♂ Anteroposterior Differential Accessbility
MAPK	EGFR core pathway: *vn, pnt*<br>Linked to EGFR pathway: *scr42A, Mtl*	*scr42A*
Wnt	Wg core pathway : *fz2*<br>Linked to Wg pathway : *Mtl*<br>Wnt5 core pathway: *drl*	*fz2*
TGF-beta	TGF-beta core pathway: *sog, dpp, Dad*	*Dad*
mTOR	mTOR core pathway: *eIF4EHP*	-
Hedgehog	Hh core pathway: *ci*	-
Notch	Notch core pathway: *N, mam, numb*	-
Hippo	Linked to hippo pathway : *ed, sdt, baz, fred, p53, Gug*	-

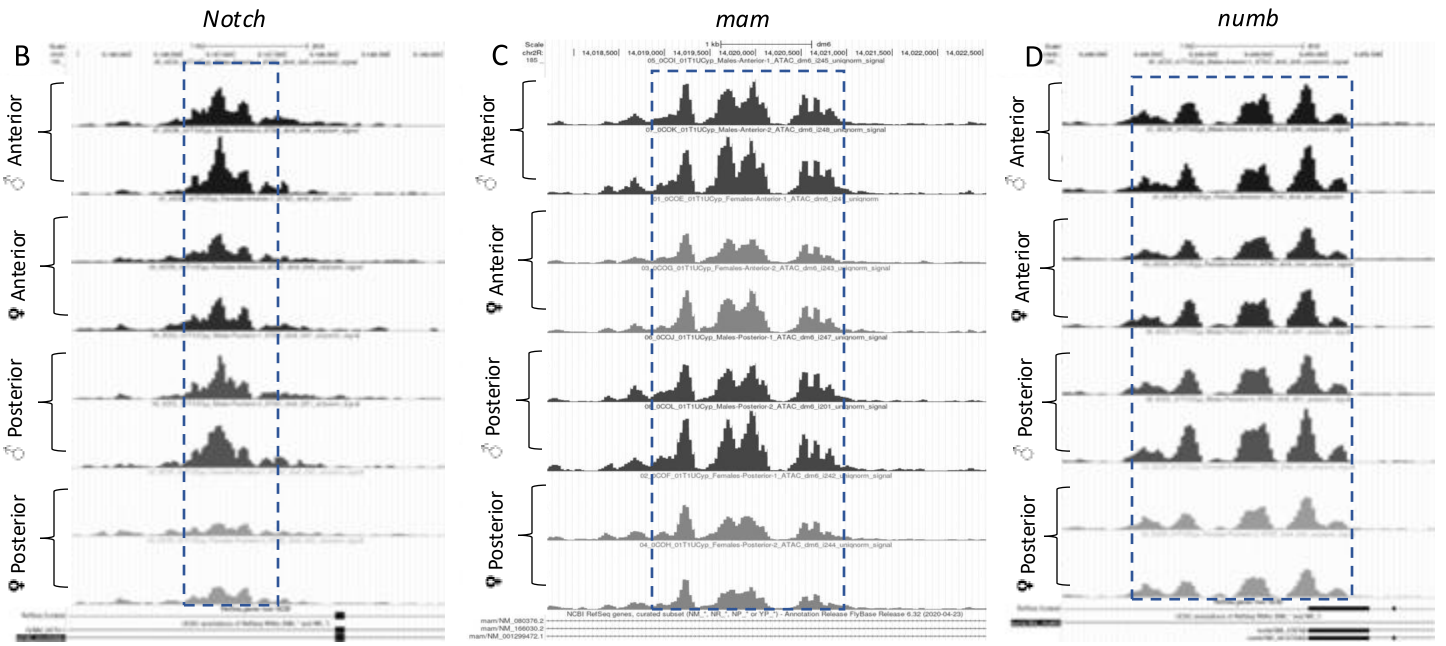

Figure S5: KEGG pathway analysis of anteroposterior differentially accessible ATAC-seq peaks, Related to Figure 4.

(A) Curated KEGG pathway analysis of anteroposterior differentially accessible ATAC-seq peaks between the anterior and posterior midgut of females and males shows enrichment in Notch (3 genes), TGF-beta (3 genes), EGFR (2 genes) and other pathways.

(B-D) ATAC-seq peaks within the *Notch* (B), *mastermind* (C) and *numb* (D) locus exhibiting reduced accessibility in the posterior female midgut compared to the anterior female or the male midgut. NCBI reference genes and their transcript structures are shown below the ATAC-seq peaks.

Figure S6

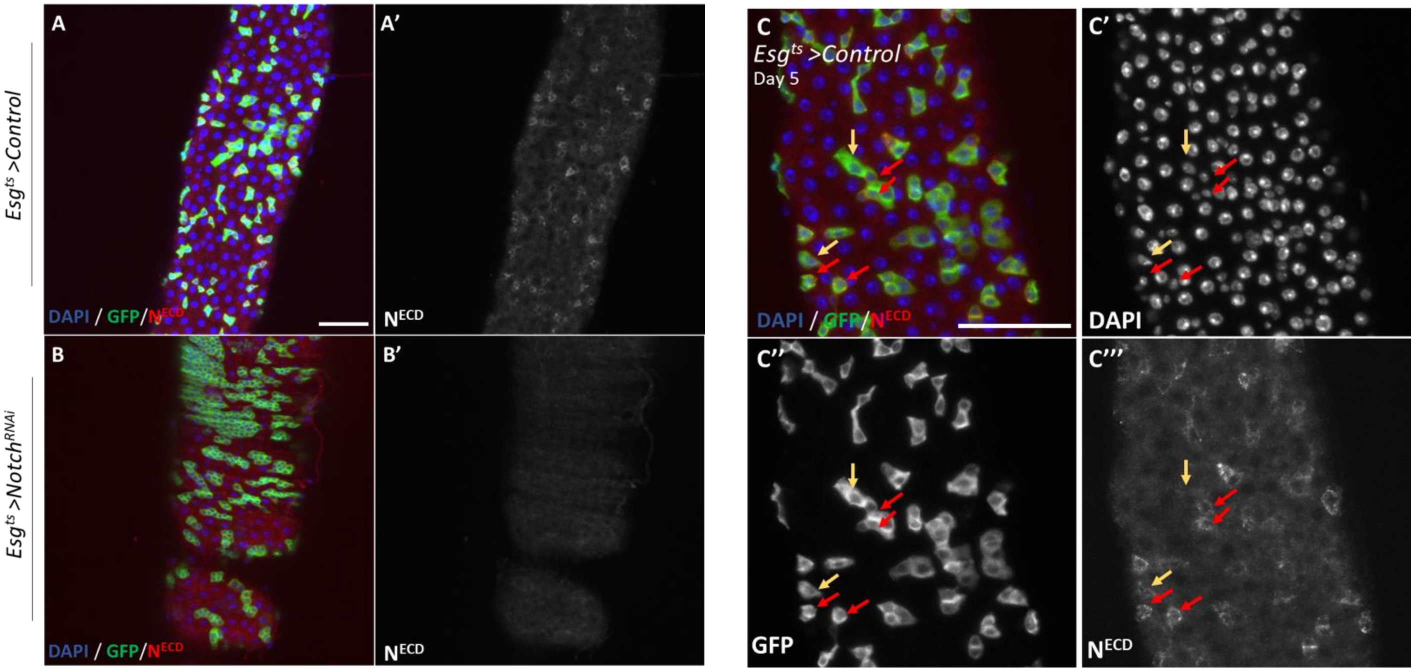

Figure S6: Variability in Notch expression in progenitor cells, Related to Figure 4.
(A and B) Female posterior midguts of *esg^{ts}-Gal4 UAS-GFP* (Esg^{ts}>control) (A-A’) and *esg^{ts}-Gal4 UAS-GFP UAS-$Notch^{RNAi}$* (Esg^{ts}>$Notch^{RNAi}$) flies (B-B) 5 days post induction at 29 ^{o}C captured in the same settings. GFP (green) marks for *esg+* cells, N^{ECD} (red) marks Notch extracellular domain-positive cells, and DAPI (blue) marks all nuclei. Magnification is 20x zoom 2. Scale bar is 50 µm. (C-C’’’) As in A at a higher magnification (40x zoom 2). Red and yellow arrows indicate progenitor cells with high and low Notch intensity, respectively. Scale bars are 75 µm.

Figure S7

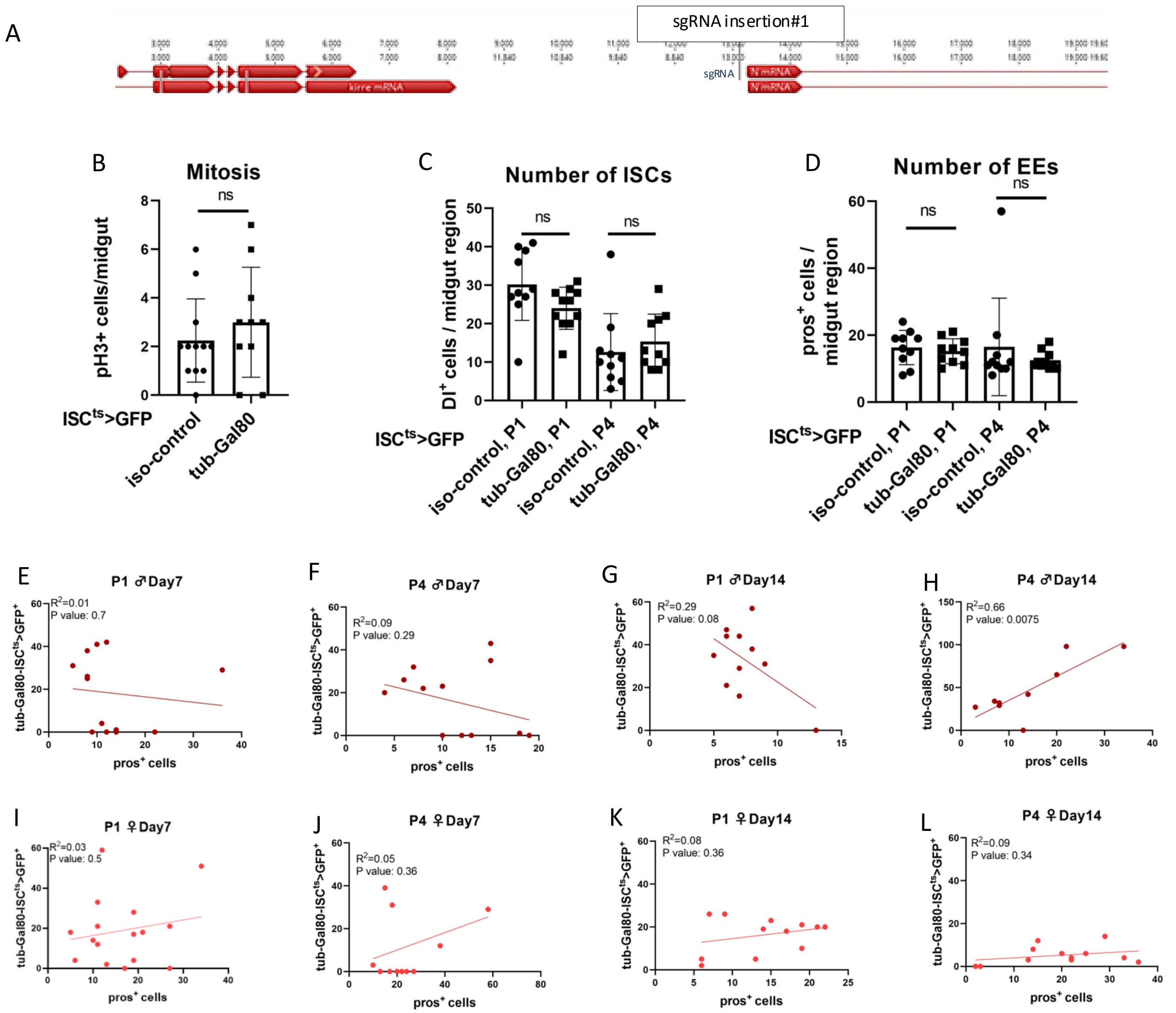

Figure S7: The *NotchTSS-331tubGal80* insertion does not affect midgut mitosis or EE numbers when crossed to *ISC*^{ts}*-Gal4 UAS-GFP*, Related to Figure 4.

(A)Validation diagram of the insertion of *tubulin-Gal80* 149bp upstream of Notch. Red structured indicate the upstream *kirre* gene and the *notch* gene, while yellow shows the side of insertion.

(B) Mitotic (pH3$^+$) cells per midgut of isogenised *tubulin-Gal80 act-Gal4 UAS-GFP* versus control *act-Gal4 UAS-GFP* males. Student's t test, n≥10 midguts.

(C) Dl$^+$ cells per region (P1 and P4) of isogenised *tubulin-Gal80 act-Gal4 UAS-GFP* versus control *act-Gal4 UAS-GFP* males. Student's t test, n≥10 midguts.

(D) Pros$^+$ cells per region (P1 and P4) of isogenised *tubulin-Gal80 act-Gal4 UAS-GFP* versus control *act-Gal4 UAS-GFP* males. Student's t test, n≥10 midguts.

(E-T) Correlation graphs between the number of GFP$^+$ cells (due to loss of TubG80, *Notch* gene) and pros$^+$ cells, marking EEs in hot spot regions, P1 and P4. (E-L) Correlation of GFP$^+$ cells and pros$^+$ cells in male (E-H) and female (I-L) midguts, on day 7 (E-F,I-J) and day 14 (G-H,K-L) using *ISC*^{ts}*-Gal4*. Linear regression (R^2), n=10-13 data points.

ns, not statistically significant.

Figure S8

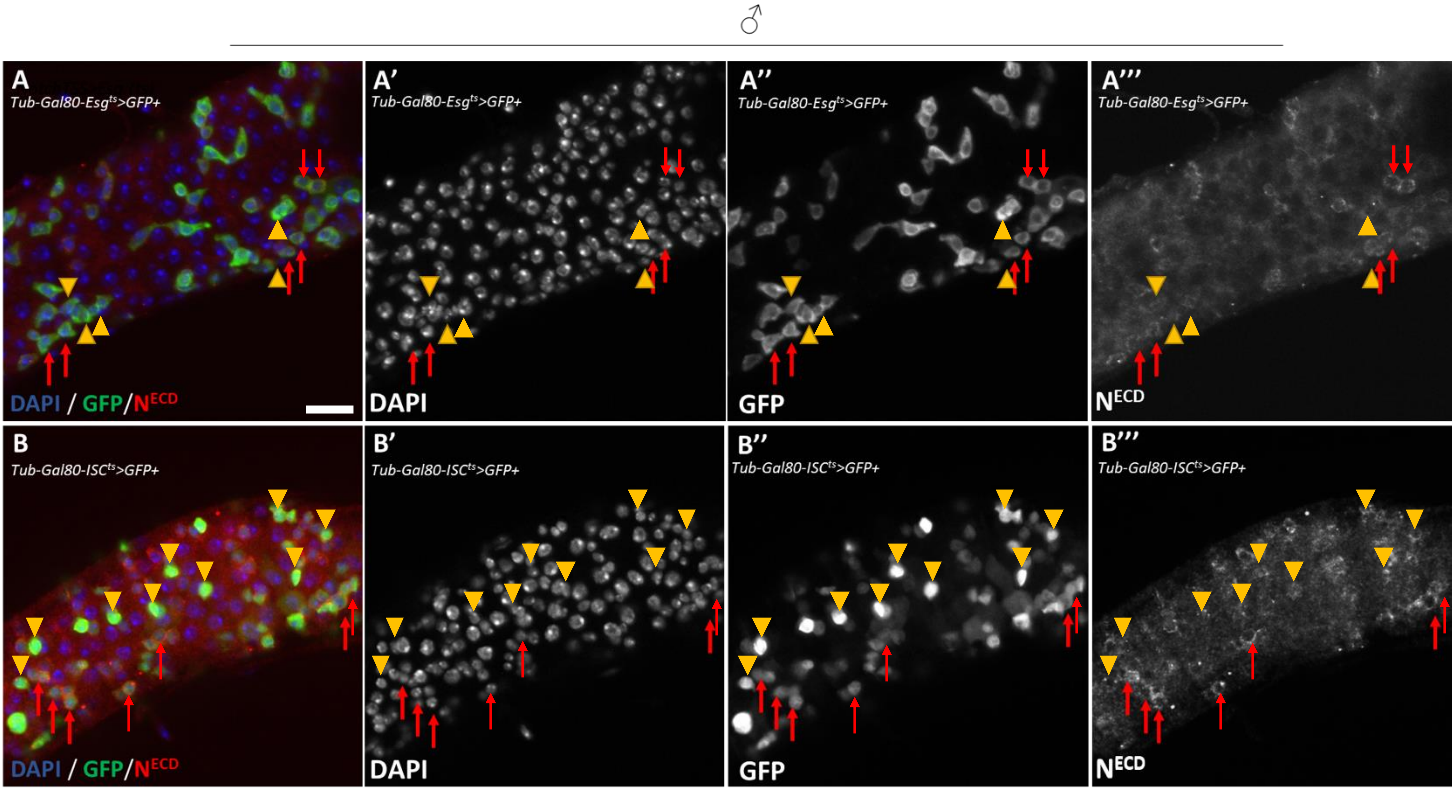

Figure S8: Variability in Notch expression in progenitor cells marked with the *NotchTSS-331tubGal80* position effect variegation tool, Related to Figure 4.

(A-B) Images of 14-days male posterior midguts derived by crossing *NotchTSS-331tubGal80* to *esg*^{ts}*-Gal4* (A-A''') or *ISC*^{ts}*-Gal4* (B-B'''). GFP (green) marks ISCs and EBs (A,A'') or ISCs only (B,B''), N^{ECD} (red) marks Notch-positive cells (A,A''', B, B''') and DAPI stains all nuclei (A,A', B, B'). Red arrows and yellow arrow heads indicate progenitor cells with high and low Notch expression, respectively. Magnification of images is 40x zoom 2. Scale bar is 50 µm.

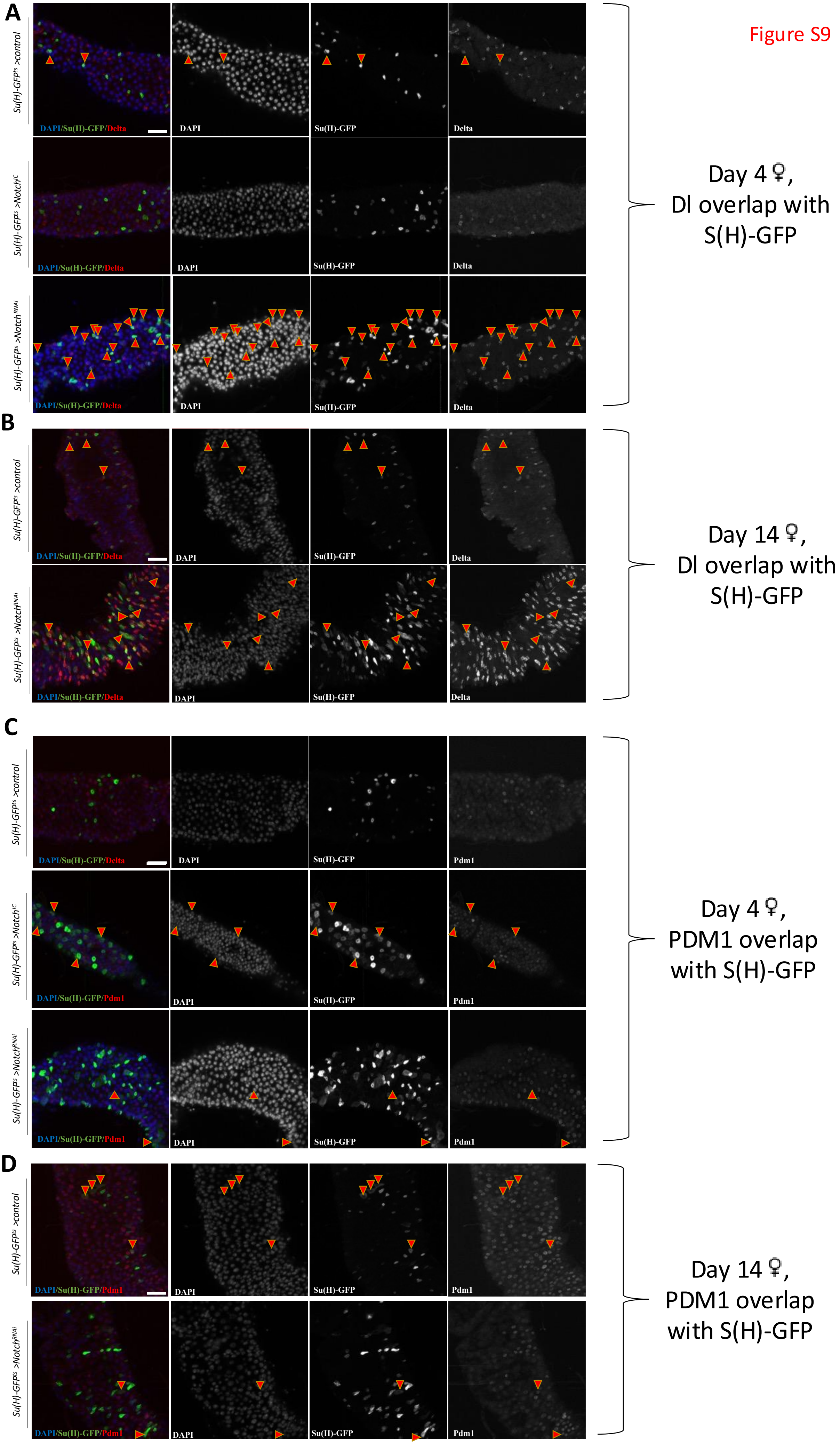
A
B
C
D
Su(H)-GFP^ts >control
Su(H)-GFP^ts >Notch^IC
Su(H)-GFP^ts >Notch^RNAi
DAPI/Su(H)-GFP/Delta
DAPI/Su(H)-GFP/Pdm1
DAPI
Su(H)-GFP
Delta
Pdm1
Day 4♀, Dl overlap with S(H)-GFP
Day 14♀, Dl overlap with S(H)-GFP
Day 4♀, PDM1 overlap with S(H)-GFP
Day 14♀, PDM1 overlap with S(H)-GFP

Figure S9

Figure S9: EBs expressing Dl or Pdm1 under three different Notch signalling levels, Related to Figure 4.

% GFP+ cells in the posterior midgut that are also Dl+ (A,B) or Pdm1+ (C,D) on day 4 (A,C) and 14 (B,D) at 29°C using female progeny of *Su(H)GBE*^{ts}*-Gal4* crossed to *UAS-Notch*^{RNAi}, *UAS-Notch*^{IC} and control *w*^{1118}. (A,B) Arrowheads indicate the abundant overlap between GFP+ and Dl+ cells in *UAS-Notch*^{RNAi} expressing flies, less so in control flies and not at all in *UAS-Notch*^{IC} expressing flies. (C,D) Arrowheads indicate the abundant overlap between GFP+ and Pdm1+ cells in control and *UAS-Notch*^{RNAi} expressing flies, and only in “ghost” EBs in *UAS-Notch*^{IC} expressing flies. GFP is lost at 14 days in *UAS-Notch*^{IC} expressing flies indicative of their fast differentiation to ECs (not shown). Scale bars are 50 µm.

Figure S10

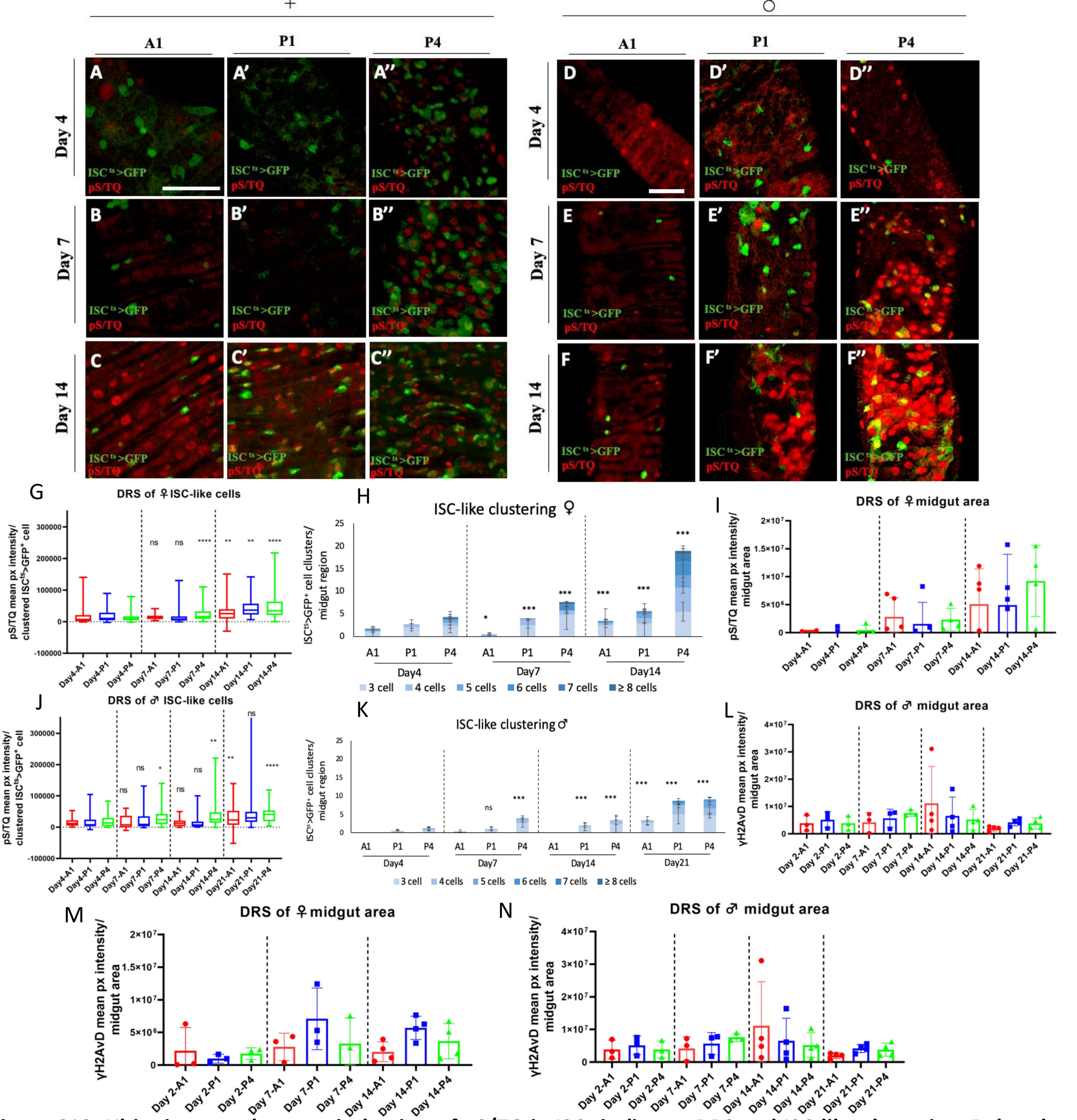

Figure S10: Ubiquitous early onset induction of pS/TQ in ISCs indicates DRS and ISC-like clustering, Related to Figure 5.

(A-F) pS/TQ signal in the A1 (A-F), P1 (A'-F') and P4 (A"-F") regions of 4- (A, D), 7- (B, E) and 14-day old (C, F) *ISC^{ts}-GFP* females (A-C) and males (D-F).

(G, J) pS/TQ mean pixel intensity per clustered *ISC^{ts}>GFP*-positive cell in the A1, P1 and P4 of 4-, 7- and 14-day old females (G) and 4-, 7-, 14- and 21-day old males (J). Student's t test, n≥6 midguts. (H-K) *ISC^{ts}>GFP*-positive cell clusters in the A1, P1 and P4 of 4-, 7- and 14-day old females (H) and 4-, 7-, 14- and 21-day old males (K). Chi-square, n=3-5 midguts. (I, L) pS/TQ fluorescence means per A1, P1 and P4 midgut area of 4-, 7- and 14-day old *ISC^{ts}>GFP* females (I) and 4-, 7-, 14- and 21-day old males (L). One-way ANOVA for multiple comparisons, n=3 midguts. (G,J,I,L) Significance compared to the corresponding region of 4-days old midguts.

(M-N) γH2AvD fluorescence means per per A1, P1 and P4 midgut area of P4 of 4-, 7- and 14-day old *ISC^{ts}>GFP* females (C) and 4-, 7-, 14- and 21-day old males (D).

Scale bars: 50 µm for (A-C,D-F). Significance is indicated by *p<0.05, **p<0.01, ***p<0.001, and ****p<0.0001; ns, not statistically significant.

Figure S11

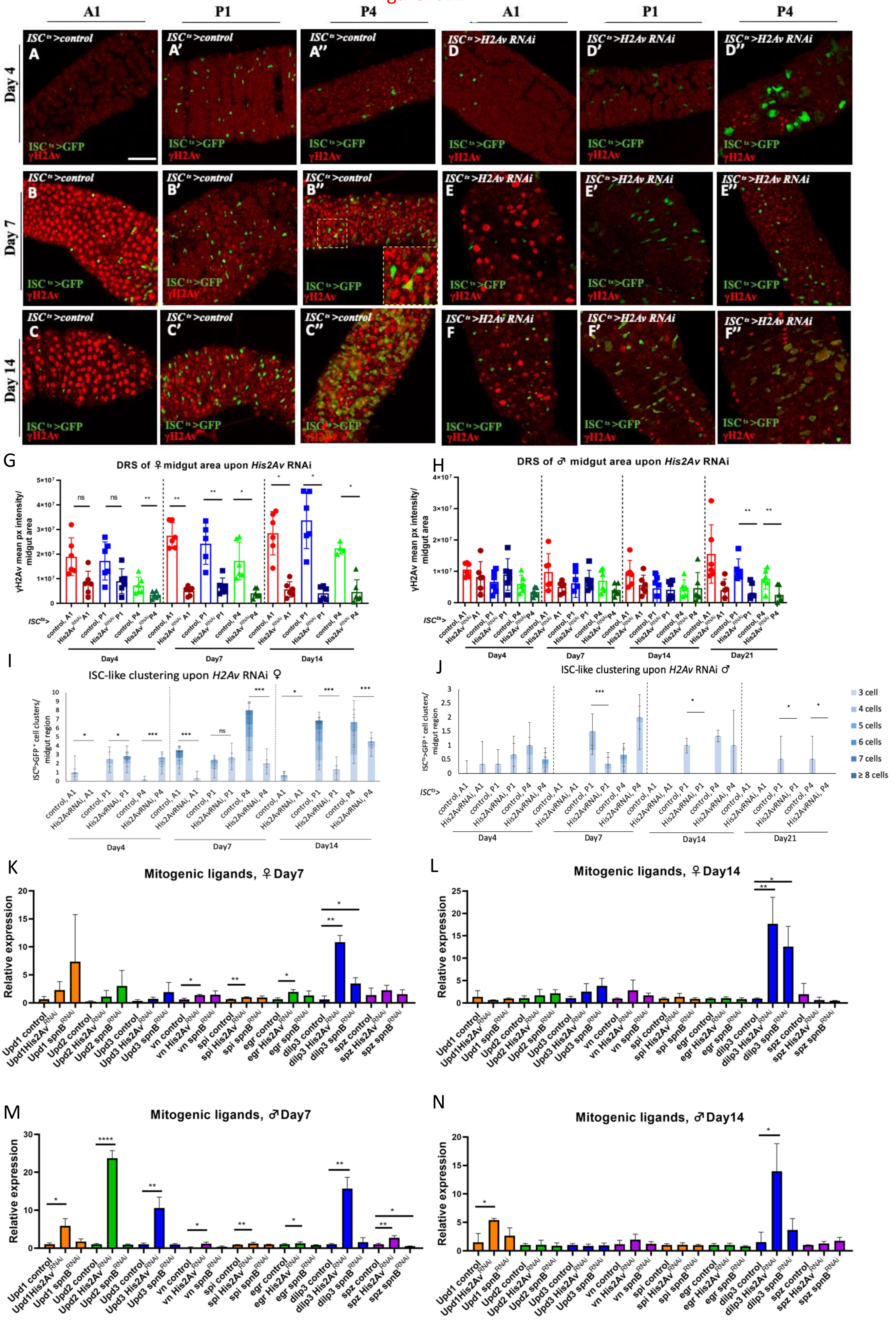
A1
P1
P4
Day 4
Day 7
Day 14
ISCts>control
ISCts>H2Av RNAi
ISCts>GFP
γH2Av
DRS of ♀ midgut area upon His2Av RNAi
DRS of ♂ midgut area upon His2Av RNAi
γH2Av mean px intensity/ midgut area
ISC-like clustering upon H2Av RNAi ♀
ISC-like clustering upon H2Av RNAi ♂
3 cell
4 cells
5 cells
6 cells
7 cells
≥ 8 cells
Day4
Day7
Day14
Day21
Mitogenic ligands, ♀ Day7
Mitogenic ligands, ♀ Day14
Mitogenic ligands, ♂ Day7
Mitogenic ligands, ♂ Day14
Relative expression

Figure S11: His2Av accelerates ISC-like clustering during aging, Related to Figure 6.

(A-F) γH2Av expression in the A1 (A-F), P1 (A'-F') and P4 (A''-F'') regions of young *ISC^{ts}-Gal4 UAS-GFP* (A-C) and *ISC^{ts}-Gal4 UAS-GFP, UAS-His2Av^{RNAi}* (D-F) females induced for 4- (A, D), 7- (B-E) and 14-days (C, F) at 29°C. (B'') Inset indicates *ISC^{ts}-Gal4 UAS-GFP* cells also expressing γH2Av.

(G-H) γH2Av mean pixel intensity per A1, P1 and P4 midgut area of young *ISC^{ts}-Gal4 UAS-GFP* versus *ISC^{ts}-Gal4 UAS-GFP, UAS-His2Av^{RNAi}* females induced for 4-, 7- and 14-days (G), and males induced for 4-, 7-, 14- and 21-days (H) at 29°C. One-way ANOVA for multiple comparisons, n=6 midguts.

(I-J) *ISC^{ts}>GFP*-positive cell clusters corresponding to the same flies described in G and H, respectively. Chi-square, n=6 midguts.

(K-N) Relative expression of a panel of mitogens in *ISC^{ts}-Gal4 UAS-GFP* female (K-L) and male (M-N) midguts co-expressing *UAS-His2Av^{RNAi}* or *UAS-spn-B^{RNAi}* for 7 (K, M) or 14 days (L, N). Mann-Whitney U test, n≥3 biological replicates.

Scale bar: 50 μm for (A-F). Significance is indicated by *p<0.05, **p<0.01, ***p<0.001, and ****p<0.0001; ns, not statistically significant.

Figure S12

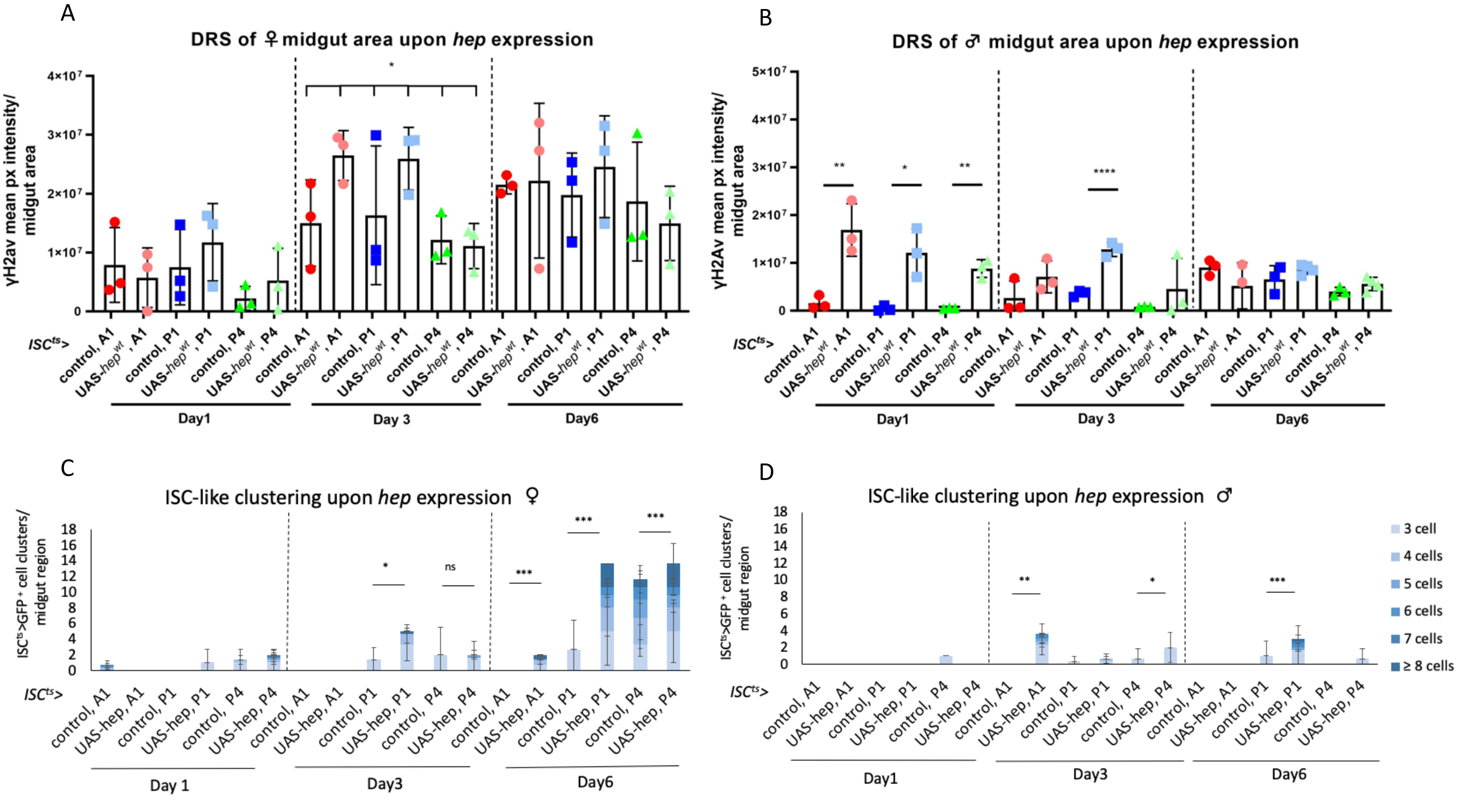

Figure S12: Hep increases DRS and ISC-like clustering, Related to Figure 7.

(A-B) γH2Av mean pixel intensity per A1, P1 and P4 midgut area of young *ISC*^{ts}*-Gal4 UAS-GFP* versus *ISC*^{ts}*-Gal4 UAS-GFP, UAS-hep* females (A) and males (B) induced for 1-, 3- and 6-days at 29°C.

(C-D) *ISC*^{ts}*>GFP*-positive cell clusters corresponding to the same flies described in C and D, respectively.

Statistical significance using data from 3 midguts via one-way ANOVA for multiple comparisons in (A-B) and chi-square test in (C-D). Significance is indicated by *p<0.05, **p<0.01, ***p<0.001, and ****p<0.0001; ns, not statistically significant.

Table S1: List of primer sequences used for generating the *NotchTSS-331tubGal80* fly line and for genes used in RT-qPCR experiments, Related to STAR Methods.

Gene Name	Gene symbol	Forward Primer	Reverse Primer
**Primers used for construction of *NotchTSS-331tubGal80* fly line via CRISPR/Cas9**			
LHA		tacccggggatccACGAAACCGAAAAT CAATTCAATTATATAC	tatcatgtctggatACTTGTAGCATTTTT TAAGTATTTTATTTTCC
RHA		aagatctccatgGCGTTTTTCAATCAAA TTTATGC3	ttacgccaagcttGATCATCTTATCTCATAG TTTTGG
PCRtest1		GAGCACTAAGAATGTGACTGCTTTC GTTTGT	ATGGGAGCAGTGGTGGAATGCC
PCRtest2		TTGCAGAGGCCAGGGCAATG	TGCGGCACAACACAGCGT
**Primers used for RT-qPCR experiments**			
Alpha tubulin	*α-tub*	GCTGTTCCACCCCGAGCAGCTGATC	GGCGAACTCCAGCTTGGACTTCTTGC
Cyclin E	*CycE*	ACAAATTTGGCCTGGGACTA	GGCCATAAGCACTTCGTCA
Delta	*Dl*	GCGACAAGCCCAATCAAT	GGTTCCAGAACGCACTCG
eiger	*egr*	AGCTGATCCCCCTGGTTTTG	GCCAGATCGTTAGTGCGAGA
Insulin-like peptide 3	*Dilp3*	CCGTTCCCTGCTGGAAAGAC	AGGCAACACTCGTCGAAGAC
puckered	*puc*	GCCACATCAGAACATCAAGC	CCGTTTTCCGTGCATCTT
Ribosomal protein L32	*RpL32*	CGGATCGATATGCTAAGCTGT	CGACGCACTCTGTTGTCG
spatzle	*spz*	GTGATTCTGGAAAATGGGATTC	TCTGTGGTGGGTGAAACTTCT
spitz	*Spi*	TGCGGTGAAGATAGCCGATC	TTCGCATCGCTGTCCCATAA
unpaired 1	*upd1*	TGCAGTTGCCGTTCTAGTCA	GCGTGGCGAATAATACTTTCC
unpaired 2	*upd2*	CGGAACATCACGATGAGCGAAT	TCGGCAGGAACTTGTACTCG
unpaired 3	*upd3*	GCAAGAAACGCCAAAGGA	CTTGTCCGCATTGGTGGT
vein	*vn*	TCACACATTTAGTGGTGGAAGC	CGTGACCTCTGCGTTCTGT

Table S2: qPCR amplification program, Related to STAR Methods.

Step	Temperature (°C)	Time (seconds)	Repeats of Cycles
Initial Denaturation	95	30	1
Denaturation	95	10	
Annealing	60	30	40
Extension	65	30	
Final extension	65	60	1
